# Distinct and Overlapping Brain Areas Engaged during Value-Based, Mathematical, and Emotional Decision Processing

**DOI:** 10.3389/fnhum.2016.00275

**Published:** 2016-06-10

**Authors:** Chun-Wei Hsu, Joshua O. S. Goh

**Affiliations:** ^1^Graduate Institute of Brain and Mind Sciences, College of Medicine, National Taiwan UniversityTaipei, Taiwan; ^2^Department of Psychology, University of PlymouthPlymouth, UK; ^3^Department of Psychology, National Taiwan UniversityTaipei, Taiwan; ^4^Center for Neurobiology and Cognitive Science, National Taiwan UniversityTaipei, Taiwan

**Keywords:** decision-making, arithmetic processing, emotion, fMRI, cognitive neuroscience

## Abstract

When comparing between the values of different choices, human beings can rely on either more cognitive processes, such as using mathematical computation, or more affective processes, such as using emotion. However, the neural correlates of how these two types of processes operate during value-based decision-making remain unclear. In this study, we investigated the extent to which neural regions engaged during value-based decision-making overlap with those engaged during mathematical and emotional processing in a within-subject manner. In a functional magnetic resonance imaging experiment, participants viewed stimuli that always consisted of numbers and emotional faces that depicted two choices. Across tasks, participants decided between the two choices based on the expected value of the numbers, a mathematical result of the numbers, or the emotional face stimuli. We found that all three tasks commonly involved various cortical areas including frontal, parietal, motor, somatosensory, and visual regions. Critically, the mathematical task shared common areas with the value but not emotion task in bilateral striatum. Although the emotion task overlapped with the value task in parietal, motor, and sensory areas, the mathematical task also evoked responses in other areas within these same cortical structures. Minimal areas were uniquely engaged for the value task apart from the other two tasks. The emotion task elicited a more expansive area of neural activity whereas value and mathematical task responses were in more focal regions. Whole-brain spatial correlation analysis showed that valuative processing engaged functional brain responses more similarly to mathematical processing than emotional processing. While decisions on expected value entail both mathematical and emotional processing regions, mathematical processes have a more prominent contribution particularly in subcortical processes.

## Introduction

Value-based decision-making in humans is thought to involve assessing costs and benefits so that options with the most incentive are chosen. Such economical choice valuations can be based either on the cognitive analysis of objective information (Lieberman, 2007), for instance being driven by numerical magnitudes, or on implicit subjective preferences that involve more affective processes (Evans, 2003), or a combination of both. It is postulated that affective processes heuristically react to problems whereas the result of cognitive deliberation of objective information may then be used to approve or modulate affective reactions (Kahneman and Frederick, 2002). Such a framework of the valuative decision process suggests that decision, cognitive, and affective operations involve distinct parallel neural networks that also overlap at certain junctures. At present, however, it is not clear whether and how the loci of brain regions involved during a value-based decision overlap with or are distinct from regions engaged during cognitive or affective processing in normal human agents.

Cognitive and affective processes have reliably been shown to engage distinct neural loci in previous studies. For example, lateral prefrontal and parietal areas are actively recruited during more cognitive processing such as in mathematical problems or magnitude representations (Menon et al., 2000; Cohen Kadosh and Walsh, 2009; Zamarian et al., 2009). Specifically, these brain areas carry out deliberative operations such as explicit comparison, manipulation, and selection of symbolic numerical information. By contrast, the medial temporal lobe, insula, and medial prefrontal cortex (MPFC) are a separate set of brain regions reported to be sensitive to affective information such as the valence of emotional facial expressions (Epstein and Pacini, 1999; Phelps and LeDoux, 2005; Lieberman, 2007). Such affective neural responses typically are triggered automatically by the presence of emotional features, do not require awareness or explicit effort, and involve more subjective valence associations or interpretations (Cannon, 1932; Foa and Kozak, 1986; Critchley et al., 2000; Anderson et al., 2003).

Experimental manipulations can bias the prominence with which cognitive or affective neural processes contribute toward a decision. For instance, higher right lateral prefrontal cortex activity is engaged when participants inhibit belief-biases and apply controlled processing to complete a logical task (Goel and Dolan, 2003). Conversely, the MPFC involved in affective processing is more active when belief-biases trumped logical thinking. In addition, greater lateral fronto-parietal activity is involved when subjects choose delayed rewards indicating more controlled reasoning about outcomes (McClure et al., 2004). By contrast, hippocampus, ventral striatal, medial frontal, as well as posterior cingulate areas are preferentially activated for immediate rewards indicating more affective contributions. These findings reflect neural correlates for how distinct cognitive and affective systems might operate when their outcomes explicitly conflict in valuative decisions. However, decisions in such contexts of cognitive-affective conflict tend to favor or emphasize one process against the other in order to meet task demands that may artificially impose a dichotomization of these neural functions. It remains difficult to specify the neural loci for whether and where the cognitive and affective systems are separate or coalesce during more unbiased valuative decisions contexts.

More normative and less biased value-based decision contexts can be instantiated when stimuli inform about magnitude and likelihood of rewards (or punishments) apart from affective information, and where mathematical operations such as the numerical calculation of expected value are not explicitly required during a task. Thus, one can either use objective information depicted by the stimuli to compute likely outcomes and arrive at a decision or one can base the decision on more subjective affective responses toward the same stimuli. We considered that under such scenarios, human decision-makers generally subscribe to objective strategies during value-based decisions that should be associated with greater reliance on cognitive mathematical computations rather than affect by default. However, to the extent that brain areas involved during affective processing overlap with regions involved in value-based decision-making, processing in these regions may potentially compete and distort cognitive mathematical computation of numerical results thus playing a role in valuative processes as well.

The aim of this study was to evaluate the involvement of neural regions engaged for cognitive and affective processes when participants made value-based decisions in an experimental context that did not explicitly bias either system. Across three decision-making tasks based on economical expected value, numerical mathematical, and face emotion information, stimuli were presented in identical visual format but with different task goals. We evaluated unique and overlap of brain regions as well as spatial correlations in functional responses across these tasks in a within-subject manner. While both cognitive and affective regions should have spatially unique and overlapping brain areas with value-based decision-making, we were particularly interested in whether and where in the brain value-based decision-making would have more in common with the mathematical or emotional task.

## Materials and methods

### Participants

Twenty right-handed normal participants from the local community (mean age (SD) 22.8 years; 11/9 males/females) completed the Value, Mathematical, and Emotion tasks in this functional magnetic resonance imaging (fMRI) study. Exclusion criteria included history or presence of neurological, psychiatric, or physical counter-indication for MRI scanning. The National Taiwan University Hospital Research Ethics Committee approved this study and all participants gave signed informed consent. All participants were remunerated for their time in the study.

### Face emotion photographs

Face stimuli used in this experiment were obtained from the Taiwan database of emotional stimuli (Liang and Chen, 2010. Retrieved from http://ssnre.psy.ntu.edu.tw/ssnre/abstract.htm) that consists of emotional facial expression photographs depicted by local professional actors using the Facial Action Coding system (Ekman and Friesen, 1977). Normative emotion ratings of each facial expression photograph were also obtained from the database such that 0 indicated least representative and 10 most representative of the specific emotional expressions. We used photographs from 24 actors (12/12 males/females) and selected their happy (mean rating (SD) 4.74 (1.46); total 40 photographs) as well as their corresponding neutral (mean rating (SD) 0.62 (0.52); total 23 photographs, one actor's neutral photograph was not available) face photographs for the fMRI decision tasks of the main study. Happy faces were used to generate 20 face pairs for the Emotion task so that each pair had a face with lower happiness rating than the other [5.55 (0.76) vs. 3.93 (1.58); *t*_(19)_ = 5.27, *p* < 0.001]. In this manner, face pairs in the Emotion task depicted different levels of emotion between the pairs across 20 trials for comparative judgment during the task. Faces in 10 pairs were of the same sex while faces in the other 10 pairs were of different sexes. Faces within each pair were always from different actors. We also used Squirlz Morph software (http://www.xiberpix.net/SqirlzMorph.html) to morph all the neutral faces into an averaged neutral face. The averaged neutral face was used in the Value and Mathematical tasks, where face emotion was not relevant, to visually control for the use of emotional faces in the Emotion task (see below).

### Decision task stimuli

**Figure 1:**
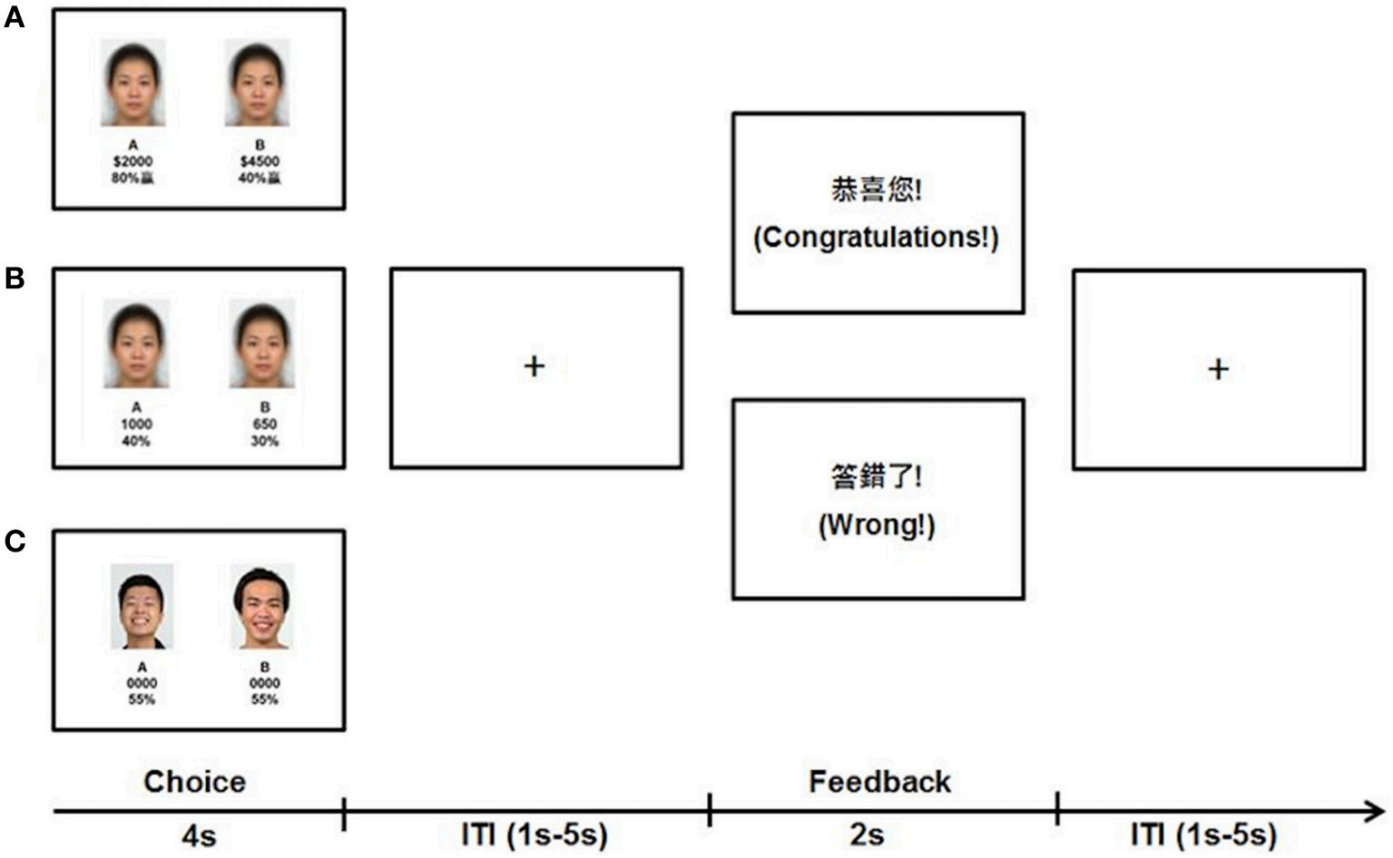
**Sample stimuli and trial timeline of the three decision tasks. (A)** In the Value task, participants decided whether they preferred the lottery in **(A,B)** based on the magnitude of money and percentage probability of obtaining the money depicted. **(B)** In the Mathematical task, participants multiplied the magnitude of points and percentages and selected the bigger product of **(A,B)**. **(C)** In the Emotion task, participants chose the person in **(A,B)** whom they felt might share more money with them.

Similar visual stimuli were used across the Value, Mathematical, and Emotional tasks with different task instructions (Figure 1). For all tasks, during the choice phase of each trial, participants saw two panels, on the left (A) and right (B) of the screen. In each panel, the top section consisted of a face stimulus bounded in a rectangular frame whereas the bottom section consisted of two numerical text items one text on top of the other. During the feedback phase, simple text indicated the outcome specific to each task. All stimuli were presented against a white background with text stimuli in black. Verbal texts were presented and instructions delivered in Mandarin, which was the first language of the participants in this Taiwan-based sample.

For the Value task, during the choice phase, the averaged neutral face was the face stimulus and used as a visual control that was not relevant to the task. For the numerical items in the panels, the top number was the magnitude of money at stake for that panel and the bottom number was the percentage probability of obtaining the magnitude specified above it. Participants were instructed to obtain as much money as possible by choosing the stakes given in the left or right panel in each trial. Participants were informed that the use of money representations in this task was only hypothetical, and remuneration was based only on time in study. To engage participants' valuative processes, we embedded five levels of difficulty across trials. In the first level, the magnitudes and probabilities across panels were congruent such that both were higher in one panel than the other. In the subsequent four incongruent levels of difficulty, all magnitudes were higher with lower probabilities in one panel compared to the other panel. Importantly, over the four incongruent levels, the numbers used were either in multiples of 5 (easier for calculation of expected value) or not (harder for calculation), and the difference of expected values between panels was either larger than 100 (more distinct difference) or smaller than 100 (less distinct difference). There were 16 trials for each difficulty level making up a total of 80 trials in the Value task. For the feedback phase, participants were shown whether they won (“Congratulations”) or not (“Wrong”) based on their choices and predetermined outcomes. To make outcomes stochastic, the predetermined outcomes were the higher expected values in 50 trials, the lower expected values in 8 trials, either of the expected values in 12 trials (participants won regardless of their choices) and neither of the expected values in 10 trials (participants loss regardless of their choices). For analysis, correct choices were defined as selecting the panel with the higher expected value.

The Mathematical task was similar to the Value task with the following exceptions. For the choice phase, the magnitude depicted points rather than money and participants were instructed to choose the panel with the larger resulting product of the top number and the bottom percentage. In addition, only three difficulty levels were embedded consisting of a congruent level, an incongruent level with smaller than 100 point difference between panels, and an incongruent level with larger than 100 points difference between panels. Manipulation of calculation difficulty (multiple of 5 or not) was not implemented for this task to avoid participants adopting random choices without invoking mathematical processes under excessive difficulty. There were 15 trials for each difficulty level making up a total of 45 trials in the Mathematical task. Feedback was then provided on whether participants answered correctly for that trial (“Correct” or “Wrong”) based on their responses and the actual arithmetic result (no predetermined outcomes).

Finally, the Emotion task was similar to the above tasks with the following exceptions. For the choice phase, the face in one panel was happier than the face in the other based on their ratings. The magnitudes and probabilities were replaced with “0000” and “55%” as fillers, respectively. Participants were instructed to decide for each trial which of the two persons they felt would share more money with them. There were a total of 20 trials in the Emotion task. Feedback was provided for each trial on whether participants chose correctly (“Congratulations”) or not (“Wrong”) based on predetermined outcomes. Predetermined outcomes were the happier face in 10 trials, less happy face in 2 trials, either of the faces in 5 trials, and neither of the faces in 3 trials. For analysis, correct choices were defined as choosing the face in each pair with the higher happiness rating.

### Brain imaging acquisition parameters

Brain imaging data were acquired using a 3T Skyra MRI scanner with a 32-channel head coil (Siemens, Erlangen, Germany). For each participant, functional images were acquired using a gradient-echo planar pulse sequence with 38 axial slices parallel to the anterior-posterior commissural plane, *TR* = 2000 ms, *TE* = 24 ms, *FA* = 90°, 64 × 64 matrix and 3.4375 × 3.4375 × 4 mm resolution. Two runs of 200, 1 run of 224 and 1 run of 108 functional volumes were acquired for the Value, Mathematical, and Emotion tasks, respectively. A T2-weighted structural image co-planar to the functional images was also obtained with 38 axial slices, *TR* = 7480 ms, *TE* = 102 ms, *FA* = 150°, 256 × 256 matrix, and 1 × 1 × 4 mm resolution. Finally, we obtained a high resolution T1-weighted structural image using a magnetization-prepared rapid gradient echo (MPRAGE) sequence with *TR* = 2000 ms, *TE* = 2.98 ms, *FA* = 9°, 192 sagittal slices, 256 × 256 matrix, and 1 × 1 × 1 mm resolution for normalization to a template space.

### fMRI decision task procedures

Stimuli for the event-related fMRI tasks were presented using E-Prime 2.0 software (Psychology Software Tools, Inc., Sharpsburg, PA, USA) and were back-projected onto a screen in the scanner room that participants viewed using a mirror mounted onto the head coil. In each run across all tasks, choice stimuli were presented on the screen for 4 s followed by the feedback stimuli for 2 s (Figure 1). Stimuli were interleaved by fixations with variable inter-trial intervals (ITI) ranging from 1 to 5 s (mean: 1.5 s). 20 s fixations preceded and ended each run to further facilitate baseline signal estimation. Participants responded using right-handed button presses with their index and middle finger (left/right panels) to indicate their choices. All participants completed the three tasks in the same order with the Value task first followed by the Mathematical then Emotion tasks to prevent biases on valuative decision processes due to preceding tasks. Trial difficulty levels and predetermined outcomes (where relevant) were presented in pseudo-random order within each run across all tasks. Prior to fMRI scan, participants underwent a practice session for the Value task outside of the scanner to familiarize with the task stimuli format and range of numbers used. Practice for the Mathematical and Emotion tasks were given before the actual runs while participants were in the scanner.

### Brain imaging data preprocessing and subject-level analysis

Brain imaging data were analyzed using SPM8 (Wellcome Trust, UK). For each participant, motion and slice-time correction was applied to the functional images, which were then coregistered to the co-planar T2 and finally T1 3D structural images. T1 images were normalized to the Montreal Neurological Institute (MNI) template using SPM8's segmentation tool. The resulting transformation parameters obtained from the segmentation were applied to the functional images to spatially normalize them to MNI space. Finally, the normalized functional images were smoothed using a 3D 8 mm FWHM Gaussian kernel.

For the subject-level fMRI analysis, we applied voxel-wise univariate general linear models (GLM) on each participant's preprocessed functional data to obtain individual whole-brain estimates of brain responses during the choice and feedback phases of the Value, Mathematical, and Emotion tasks. For the Value task, the GLM included 15 regressors based on the trial onsets for 5 difficulty levels of the choice, gain feedback, and no-gain feedback trials. Similarly for the Mathematical task, there were 12 regressors based on 3 difficulty levels of the choice and feedback of correct and incorrect trials. For the Emotion task, there were 3 regressors based on the choice phase, gain feedback, and no-gain feedback trials. Onset delta functions were convolved with the canonical hemodynamic response function (HRF) and six additional covariate motion parameters also included in the GLMs. Contrasts were generated to capture the mean neural responses across difficulty levels for each individual participant for the Value, Mathematical, and Emotion tasks, which were then used in subsequent group-level analyses. Specifically, for each task, we computed voxel-wise contrasts of the average choice phase regression coefficients estimating neural responses over all difficulty levels relative to baseline as well as the average feedback phase regression coefficients across gain and no-gain (or correct and incorrect) outcomes relative to baseline. Note that subject-level contrasts were weighted to account for the different number of runs contributing to the voxel-wise mean response estimates for each task. In addition, only correct trials were included in the analysis for the Mathematical task.

For the group-level analysis, we applied a factorial model with Task as the independent variable with three levels: Value, Mathematical, and Emotion. We then generated group-level voxel-wise contrast t-maps for each task relative to baseline. These contrast t-maps were then submitted to further conjunction analyses.

### Conjunction analyses

Conjunction analyses were conducted using in-house scripts with Matlab version 8.0.0.783 (R2012b) (MathWorks, Inc., Natick, Massachusetts, USA) to identify brain regions where significant neural responses during the Value, Mathematical, and Emotion tasks overlapped and where they were unique to each task. We used the minimal t-statistic approach (Nichols et al., 2005) for all conjunctions with the voxel threshold set at *p* < 0.001 uncorrected and additionally applied a cluster size threshold of 50 voxels. These criteria fulfilled a whole-brain alpha of *p* < 0.001 using Monte Carlo simulation with 10,000 iterations (Slotnick et al., 2003). Using this approach, we identified regions fulfilling the following seven conjunctions: (1) Value ∩ Mathematical ∩ Emotion, (2) Value ∩ Mathematical ∩ ~Emotion, (3) Value ∩ ~Mathematical ∩ Emotion, (4) ~Value ∩ Mathematical ∩ Emotion, (5) Value ∩ ~Mathematical ∩ ~Emotion, (6) ~Value ∩ Mathematical ∩ ~Emotion, and (7) ~Value ∩ ~Mathematical ∩ Emotion.

To further validate and quantify the neural response patterns in brain areas involved across the three tasks, we defined regions-of-interest (ROIs) based on the above conjunction analyses. Specifically, ROIs were the separate suprathreshold functional clusters identified from the above seven conjunction criteria applied on the minimal t-statistic brain images. Note that we excluded the visual, somatosensory, and motor areas since it is not surprising that there would be common neural responses in these brain regions across tasks due to the identical stimuli format and motor response requirements. In addition, voxels that passed a more exclusive conjunction criterion (e.g., ~Value ∩ ~Mathematical ∩ Emotion) but had a spatial distribution that appeared to be in the immediate periphery of more principal clusters of more inclusive conjunctions (e.g., Value ∩ Mathematical ∩ Emotion) were regarded as reflecting residual smoothing of neural responses and were also excluded. From the resulting ROIs that met requirements, mean choice and feedback phase neural response estimates across voxels within the ROIs for each task were extracted for each individual and submitted to external statistical analyses.

### Spatial correlation analysis

We performed a spatial correlation analysis to quantify the degree of similarity of whole-brain voxel-wise responses during the choice and feedback phases of the Value, Mathematical, and Emotion tasks. For the spatial correlation between Value and Mathematical tasks, for each participant, we first extracted choice or feedback phase responses from voxels with significant responses during the Value and Mathematical tasks within a gray matter mask (obtained from the segmentation step; set at 0.8 intensity threshold). We then applied Pearson's correlation on these voxel responses between the two tasks and transformed the resulting correlation coefficients into z-scores via Fisher Z transform. This spatial correlation analysis captures the correspondence of voxel-wise patterns of neural responses regardless of the magnitude of responses within each voxel. As such, it avoids issues related to more direct comparisons of neural responses between tasks that may be biased due to task difficulty or other baseline differences. Identical steps were applied for the spatial correlations between the Value and Emotion tasks and for feedback phase data accordingly. The resulting z-scores indexing each participant's whole-brain functional similarity between the Value and Mathematical tasks and between the Value and Emotion tasks were then compared using paired *t*-tests.

### A priori anatomical ROI analysis

To complement the above more data-driven approach to evaluate responses in ROIs from the conjunction analysis, we also examined neural responses in ROIs reported in previous meta-analyses to be involved in value-based, mathematical, and emotional processing. This approach identifies and evaluates neural responses in these brain areas in a manner that is independent of task-related functional responses in our experiment. Value-based ROIs were defined in the striatum (L: −12, 12, −6; R: 12, 10, −6) and medial frontal cortex (L: −2, 40, −8; R: 2, 46, 08) (see Bartra et al., 2013), mathematical processing ROIs in the lateral frontal (L: −42, 4, 40; R: 46, 10, 28), superior parietal lobule (L: −26, −60, 46; R: 30, −62, 44), and inferior parietal lobule (L: −44, −40, 42; R: 38, −46, 42) (Arsalidou and Taylor, 2011), and emotion processing ROIs in the middle temporal gyrus (L: −56, −58, 4; R: 56, −44, 4), insular cortex (L: −26, 20, −4; R: 42, 10, 12), amygdala (R: 25, −1, −17), and anterior cingulate cortex (L: −6, 36, 22) (Fusar-Poli et al., 2009; Brooks et al., 2012). All these *a priori* ROIs were constructed as 5 mm radii spheres centered around the respective reported MNI coordinates. Choice and feedback response estimates were then extracted from these ROIs as above and submitted to external statistical comparisons.

## Results

### Behavioral performance

Behavioral accuracies for the Value and Mathematical tasks showed satisfactory performance in all participants across tasks (see Supplementary Table 1 and Supplementary Figures 1–3 for performances broken down by difficulty level). A one-way repeated-measures ANOVA yielded a significant difference across tasks [*F*_(2, 57)_ = 23.97, *p* < 0.001]. Tukey *post-hoc* tests revealed that accuracy was higher for the Mathematical task [mean (SD): 78.1 (7.0) %), *p* = 0.001] compared with the Value [mean (SD): 63.4 (5.4) %] and Emotion [mean (SD): 56.8 (14.3) %] tasks. Accuracies for the Value and Emotion tasks were not significantly different (*p* = 0.100).

For response times, a one-way repeated-measures ANOVA yielded a significant difference across tasks [*F*_(2, 57)_ = 18.1, *p* < 0.001]. Tukey *post-hoc* tests revealed that response times were significantly faster for the Value [mean (SD): 1982 (436) ms, *p* = 0.001] and Emotion [mean (SD): 1759 (375) ms, *p* < 0.001] tasks compared with the Mathematical task [mean (SD): 2447 (280) ms]. Response times in the Value and Emotion tasks were not significantly different (*p* = 0.145). Thus, whereas the Mathematical task might generally be more difficult than the Value and Emotion tasks, our data suggest that the Value task was no more difficult than the Emotion task.

### Choice phase whole-brain conjunction analysis

**Figure 2:**
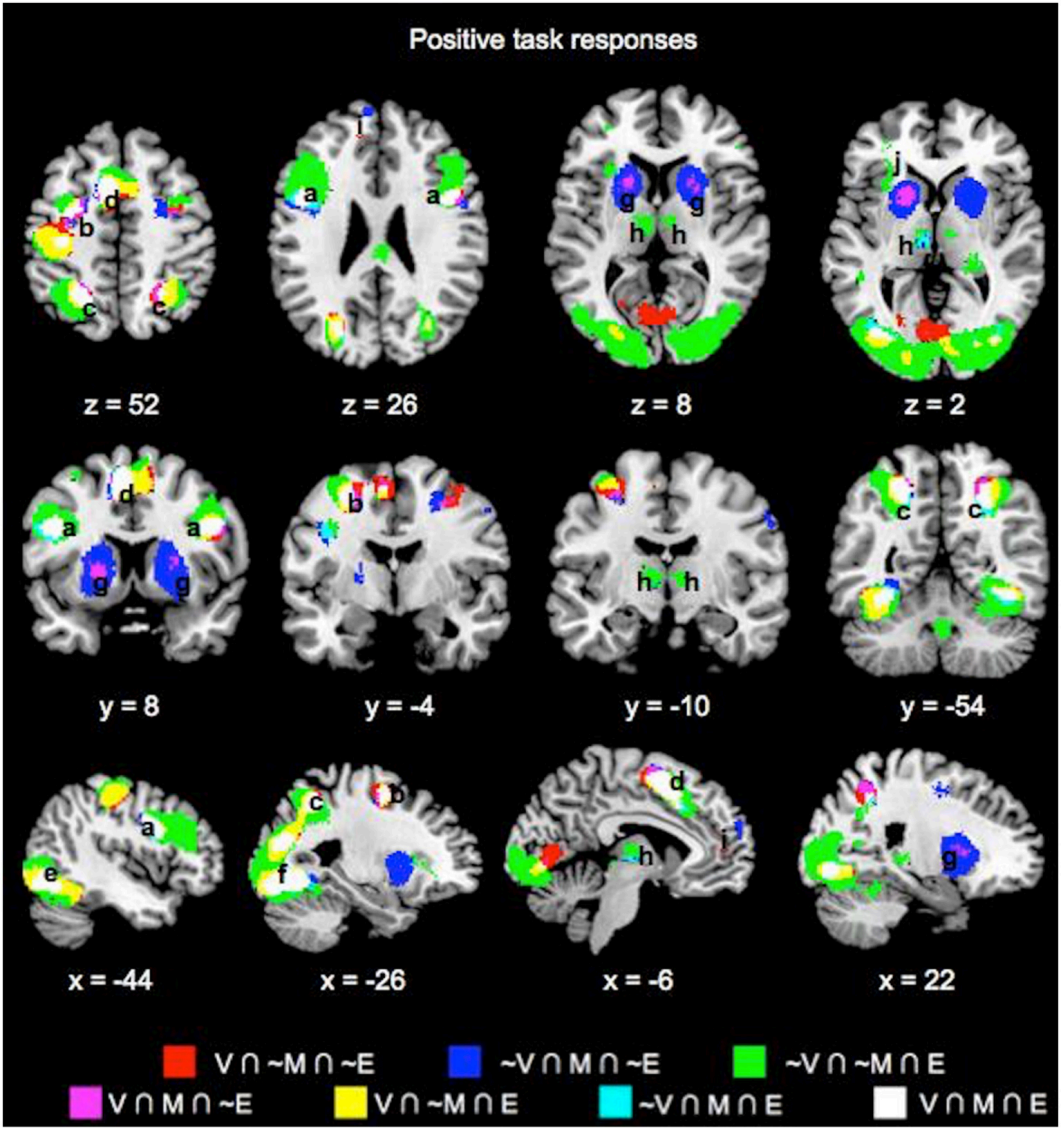
**Overlay maps showing key brain regions with significant positive task responses during the choice phases of the Value (V), Mathematical (M), and Emotion (E) tasks, as well as their overlapping areas**. Region labels: (a) Bilateral Inferior Frontal Gyri, (b) L Middle Frontal Gyrus, (c) Bilateral Superior/Inferior Parietal Lobules, (d) Bilateral Supplementary Motor Areas, (e) Bilateral Occipital areas, (f) L Fusiform Gyrus, (g) Bilateral Putamen, (h) Bilateral Thalamus, (i) Bilateral Anterior Medial Superior Frontal Gyri, (j) L Insula. Voxel significance threshold was set at *p* < 0.001 with cluster size 50.

Table 1 lists the peak coordinates and responses from the formal conjunction analyses of the mean choice phase whole-brain responses. Figure 2 further illustrates the key common and unique brain regions showing significant positive task responses during the choice phases of the Value, Mathematical, and Emotion tasks. As seen from Table 1 and Figures 2A–F, significant task-positive brain responses were present across all three tasks in the lateral frontal, parietal, supplementary motor, motor, somatosensory, and visual areas. Importantly, the Value and Mathematical tasks additionally showed common engagement of the bilateral putamen, which was minimally responsive during the Emotion task (Figure 2G). Moreover, the Value and Emotion tasks elicited common significant voxels in parietal, supplementary motor, motor, somatosensory, and visual areas that were not responsive during the Mathematical task. However, we note that these significant voxels common to Value and Emotion but not Mathematical tasks were located in the same brain structures that were commonly engaged across all three tasks, albeit occupying different areas of those structures (see Supplementary Figures 1–3 for replication of these findings using a more balanced number of trials across tasks involving the easiest conditions).

**Table 1:** **Peak MNI coordinates with Brodmann's Areas (BA) and minimal t-statistic of conjunction analyses of brain regions with significant common or unique responses during the choice phases of the Value (V), Mathematical (M), or Emotion (E) tasks**.

**Conjunction**	**Regions**	**BA**	***x***	***y***	***z***	***t***
V ∩ M ∩ E	L Inferior Frontal Gyrus Pars Opercularis	44	−45	9	27	4.95
	R Inferior Frontal Gyrus Pars Opercularis	44	45	8	29	5.36
	L Middle Frontal Gyrus	6	−26	−3	53	4.27
	L Supplementary Motor Area	6	−8	6	54	6.29
	L Precentral Gyrus	6	−44	2	33	5.42
	L Postcentral Gyrus	4	−36	−24	53	4.23
	L Superior Parietal Lobule	7	−22	−54	49	5.16
	R Superior Parietal Lobule	7	24	−54	45	4.57
	L Fusiform Gyrus	18	−26	−74	−8	7.02
	R Fusiform Gyrus	37	38	−44	−20	5.02
	R Lingual Gyrus	18	18	−84	−8	5.08
	L Middle Occipital Gyrus	19	−29	−74	21	4.69
	L Inferior Occipital Gyrus	19	−33	−81	−11	6.58
	R Inferior Occipital Gyrus	19	39	−75	−9	5.81
V ∩ M ∩~E	L Supplementary Motor Area	6	−6	0	65	4.32
	R Superior Parietal Lobule	7	24	−60	53	3.91
	L Putamen	−	−18	12	2	4.92
	R Putamen	−	26	9	11	4.12
V ∩~M ∩ E	L Supplementary Motor Area	6	−6	15	56	4.23
	R Supplementary Motor Area	32	2	9	54	4.81
	L Precentral/Postcentral Area	3	−38	−27	51	5.95
	L Inferior Parietal Lobule	7	−29	−56	47	4.36
	R Inferior Parietal Lobule	7	32	−54	48	5.24
	R Fusiform Gyrus	37	39	−44	−26	4.84
	L Middle Occipital Gyrus	18	−15	−89	−3	5.35
	R Middle Occipital Gyrus	19	32	−78	21	4.21
	L Inferior Occipital Gyrus	19	−42	−84	−5	5.79
	L Supramarginal Gyrus	40	−62	−33	33	−4.58
~V ∩ M ∩ E	L Precentral Gyrus	6	−39	−3	30	5.22
	R Fusiform Gyrus	37	38	−44	−14	5.18
	L Thalamus	−	−6	−15	2	4.26
V ∩~M ∩~E	R Precentral Gyrus	6	39	−5	57	4.10
	L Supplementary Motor Area	6	−9	−5	61	4.67
	L Precentral Gyrus	6	−32	−17	57	4.66
	L Lingual Gyrus	17	−5	−71	6	4.68
	R Lingual Gyrus	17	5	−74	3	4.37
	R Dorsal Superior Medial Frontal Gyrus	8	3	32	51	−4.66
	L Inferior Frontal Gyrus Pars Triangularis	48	−50	17	7	−4.67
	L Supramarginal Gyrus	48	−60	−24	21	−4.40
	R Supramarginal Gyrus	48	60	−24	31	−4.63
	R Angular Gyrus	39	46	−57	40	−3.98
~V ∩ M ∩~E	L Anterior Superior Medial Frontal Gyrus	10	−6	60	26	4.28
	R Anterior Superior Medial Frontal Gyrus	10	5	63	21	3.65
	R Precentral Gyrus	6	24	−3	51	4.37
	R Supplementary Motor Area	6	5	0	66	4.18
	L Lingual Gyrus	19	−29	−56	−5	5.53
	L Putamen	−	−20	15	9	7.70
	R Putamen	−	20	9	6	8.51
	L Insula	48	−42	18	0	−4.91
~V ∩~M ∩ E	L Inferior Frontal Gyrus Pars Orbitalis	47	−30	35	−3	4.91
	L Inferior Frontal Gyrus Par Opercularis	44	−45	14	32	6.69
	R Inferior Frontal Gyrus Pars Opercularis	44	50	15	32	7.66
	L Inferior Frontal Gyrus Pars Triangularis	48	−44	23	26	7.50
	R Inferior Frontal Gyrus Pars Triangularis	48	47	27	24	6.09
	L Middle Cingulate Gyrus	32	−5	23	39	5.27
	R Middle Cingulate Gyrus	23	6	−23	30	5.59
	L Supplementary Motor Area	8	−6	21	48	6.97
	R Supplementary Motor Area	6	3	14	56	5.48
	L Precentral Gyrus	6	−42	6	36	6.24
	L Postcentral Gyrus	4	−44	−23	62	4.49
	L Inferior Parietal Lobule	7	−32	−56	48	5.95
	R Inferior Parietal Lobule	40	38	−56	51	4.95
	R Fusiform Gyrus	37	33	−51	−6	7.15
	R Lingual Gyrus	18	9	−83	−9	7.04
	R Calcarine Sulcus	18	18	−89	2	7.23
	L Insula	48	−30	18	3	5.54
	L Thalamus	−	−8	−17	8	5.10
	R Thalamus	−	6	−11	5	4.91
	R Postcentral Gyrus	2	26	−39	62	−4.99

L, Left Hemisphere; R, Right Hemisphere.

We also note the expansive area where Emotion task responses were unique. Specifically, unique Emotion task voxels in the parietal and visual areas extended into the periphery of regions commonly engaged for the three tasks (Figures 2C,E,F). Unique Emotion task voxels in the lateral frontal and supplementary motor areas extended more anteriorly with Value and Mathematical tasks overlapping in more posterior portions of these regions (Figures 2A,D). Interestingly, the Emotion tasks engaged the bilateral thalamus with a part of the left thalamus in common with the Mathematical but not the Value task (Figure 2H). Apart from this area, the left precentral, and right fusiform gyri, other areas where the Mathematical and Emotion tasks overlapped were always in common with the Value task. Only a region extending anteriorly in bilateral lingual gyri and in the periphery of voxel clusters common to all three tasks in supplementary motor and motor areas showed significant voxels unique to the Value and not Mathematical or Emotion tasks. Finally, the Mathematical task uniquely evoked significant neural responses in bilateral superior medial frontal gyrus but not the Value or Emotion tasks (Figure 2I).

**Figure 3:**
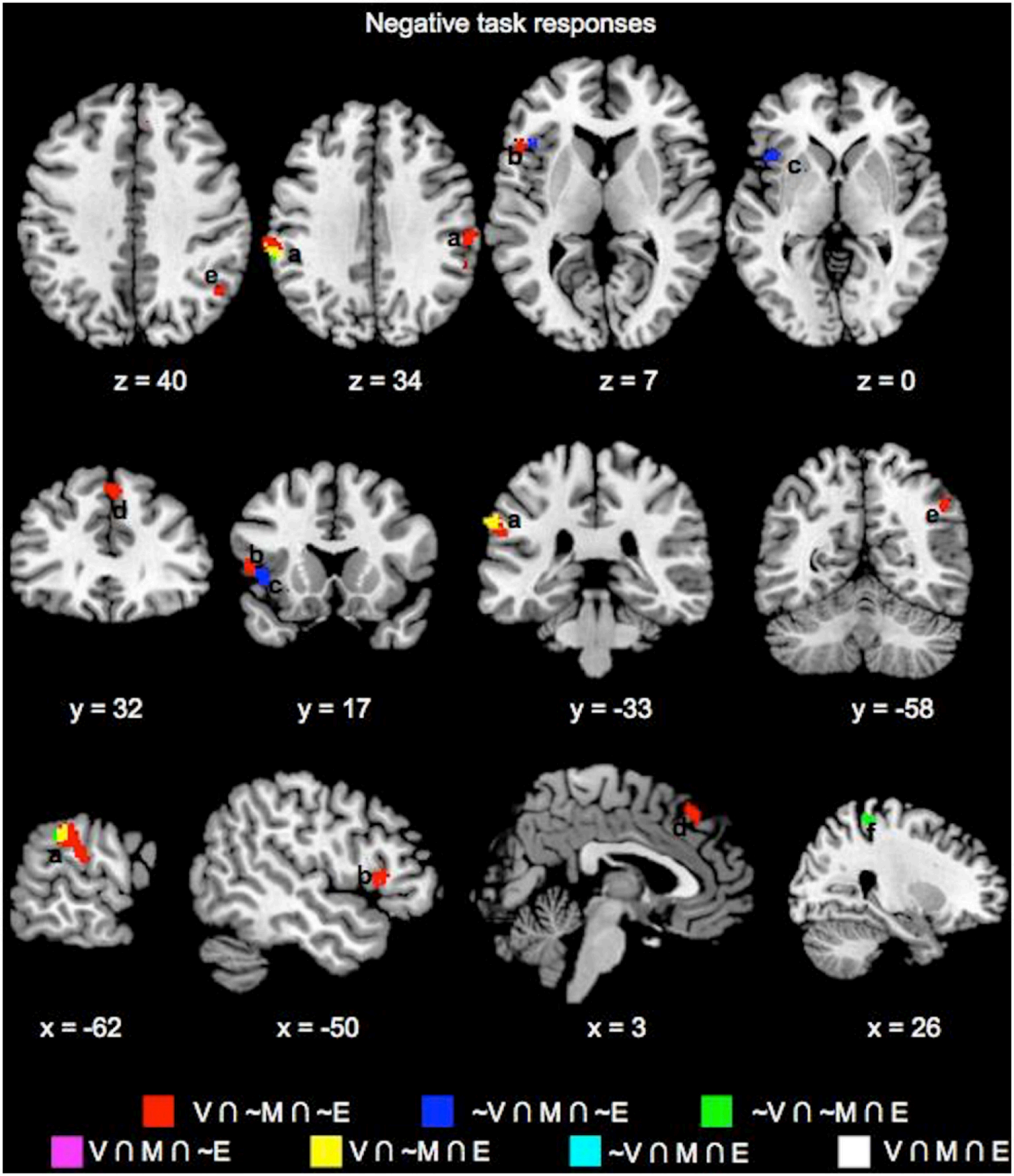
**Overlay maps showing key brain regions with significant negative task responses during the choice phases of the Value (V), Mathematical (M), and Emotion (E) tasks, as well as their overlapping areas**. Region labels: (a) Bilateral Supramarginal Gyrus, (b) L Inferior Frontal Gyrus, (c) L Insula, (d) L Dorsal Medial Superior Frontal Gyrus, (e) R Angular Gyrus, (f) R Postcentral Gyrus. Voxel significance threshold was set at *p* < 0.001 with cluster size 50.

Key common and unique brain regions showing significant negative task responses during the choice phases of the three tasks are also listed in Table 1 and shown in Figure 3. The main regions showing task-negative responses were the bilateral supramarginal gyrus, left inferior frontal gyrus, insula, right superior medial frontal, postcentral and angular gyri. Of these, only the left supramarginal gyrus showed significant overlap between the Value and Emotion tasks (Table 1; Figure 3B).

### Choice phase ROI analysis

**Figure 4:**
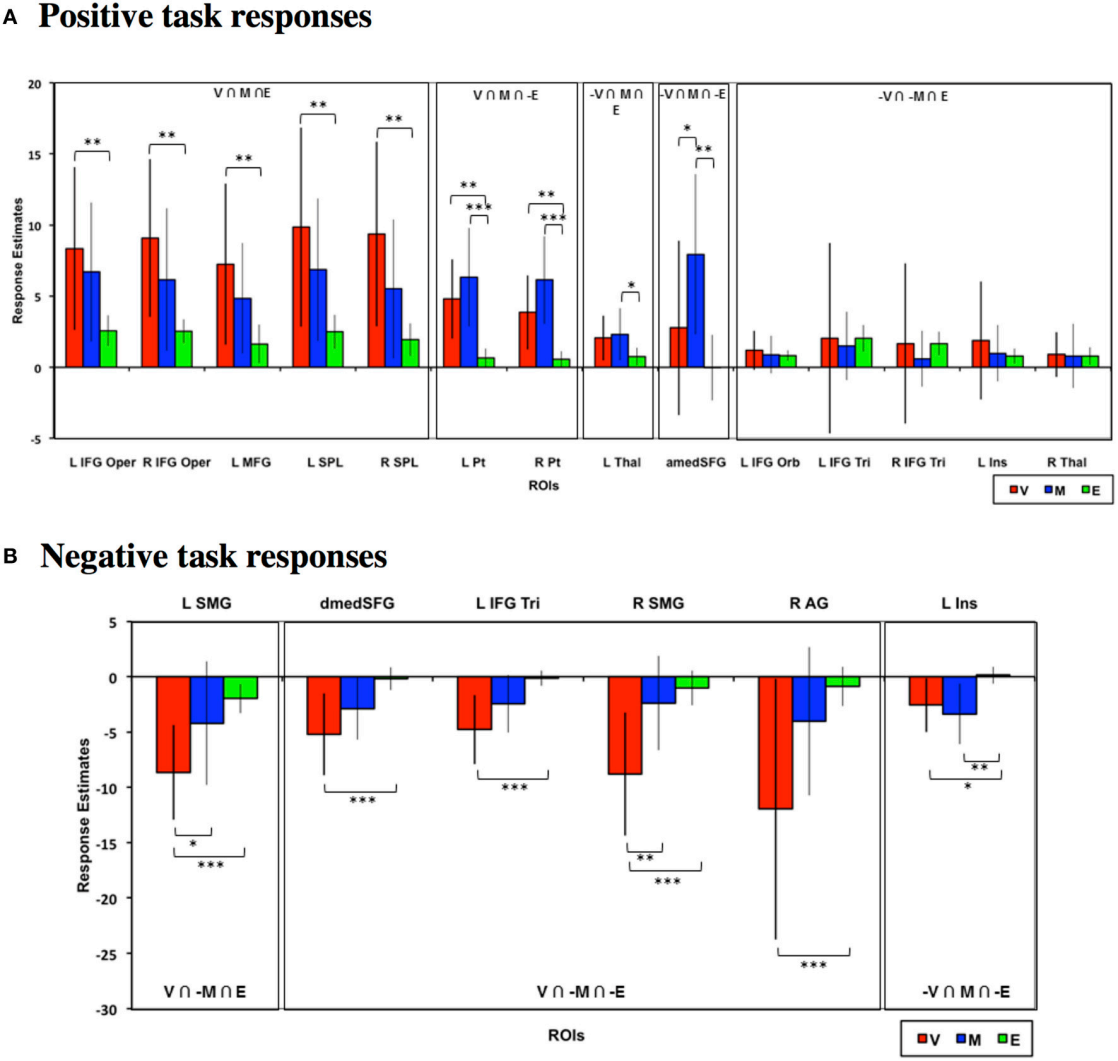
**Mean estimated neural responses during the choice phases of the Value (V), Mathematical (M), and Emotion (E) tasks in ROIs based on the conjunction analyses**. Error bars denote 95% confidence intervals. Plots are shown for ROIs showing **(A)** Positive and **(B)** negative task responses (^*^p < 0.05, ^**^p < 0.01, ^***^p < 0.001). Region labels: L IFG Oper, Left Inferior Frontal Gyrus Pars Opercularis; R IFG Oper, Right Inferior Frontal Gyrus Pars Opercularis; L MFG, Left Middle Frontal Gyrus; L SPL, Left Superior Parietal Lobule; R SPL, Right Superior Parietal Lobule; L Pt, Left Putamen; R Pt, Right Putamen; L Thal, Left Thalamus; amedSFG, Anterior Medial Superior Frontal Gyrus; dmedSFG, Dorsal Medial Superior Frontal Gyrus; L IFG Orb, Left Inferior Frontal Gyrus Pars Orbitalis; L IFG Tri, Left Inferior Frontal Gyrus Pars Triangularis; R IFG Tri, Right Inferior Frontal Gyrus Pars Triangularis; L Ins, L Insula (for positive task response: −30, 18, 3; for negative task responses: −42, 18, 0), R Thal, Left Thalamus; L SMG, Left Supramarginal Gyrus; R SMG, Right Supramarginal Gyrus; R AG, Right Angular Gyrus.

To validate and quantify the overlap patterns from the whole-brain analysis, we defined functional ROIs based on the whole-brain conjunction analyses above as listed in Table 1 (see Materials and Methods for full criteria) and examined the choice phase neural responses across tasks in these ROIs. Figure 4 depicts the neural responses across these ROIs that showed either significant task effects relative to baseline or significant task differences. As seen in Figure 4A, frontal, parietal, and striatal ROIs that evinced significant positive task responses during both Value and Mathematical tasks (Value ∩ Mathematical ∩ Emotion; Value ∩ Mathematical ∩ ~Emotion) showed significantly higher neural responses during the Value compared to the Emotion tasks. In addition, the Value task also evoked significantly lower, more suppressed, responses in all ROIs with negative task responses (Figure 4B). The Mathematical task evoked significantly higher neural responses than the Emotion task in bilateral putamen, medial superior frontal, and left thalamus ROIs showing positive task responses and more suppressed responses in the left insula ROI located at (−42, 18, 0) showing negative task responses. The Mathematical task also evoked significantly higher responses than the Value task in medial superior frontal ROI. By contrast, the Value task induced significantly more suppression than the Mathematical task in bilateral supramarginal ROIs. In all other ROIs, Value and Mathematical task responses were not significantly different. Responses during the Emotion task were the smallest in magnitude relative to the other two tasks and significance of responses relative to baseline were largely due to the smaller variability of Emotion task neural responses across participants. This was particularly the case for the bilateral inferior frontal, left insula (at −30, 18, 3; compare this to the more lateral insula ROI above), and thalamus^1^ ROIs in Figure 4A.

### Choice phase spatial correlation analysis

**Figure 5:**
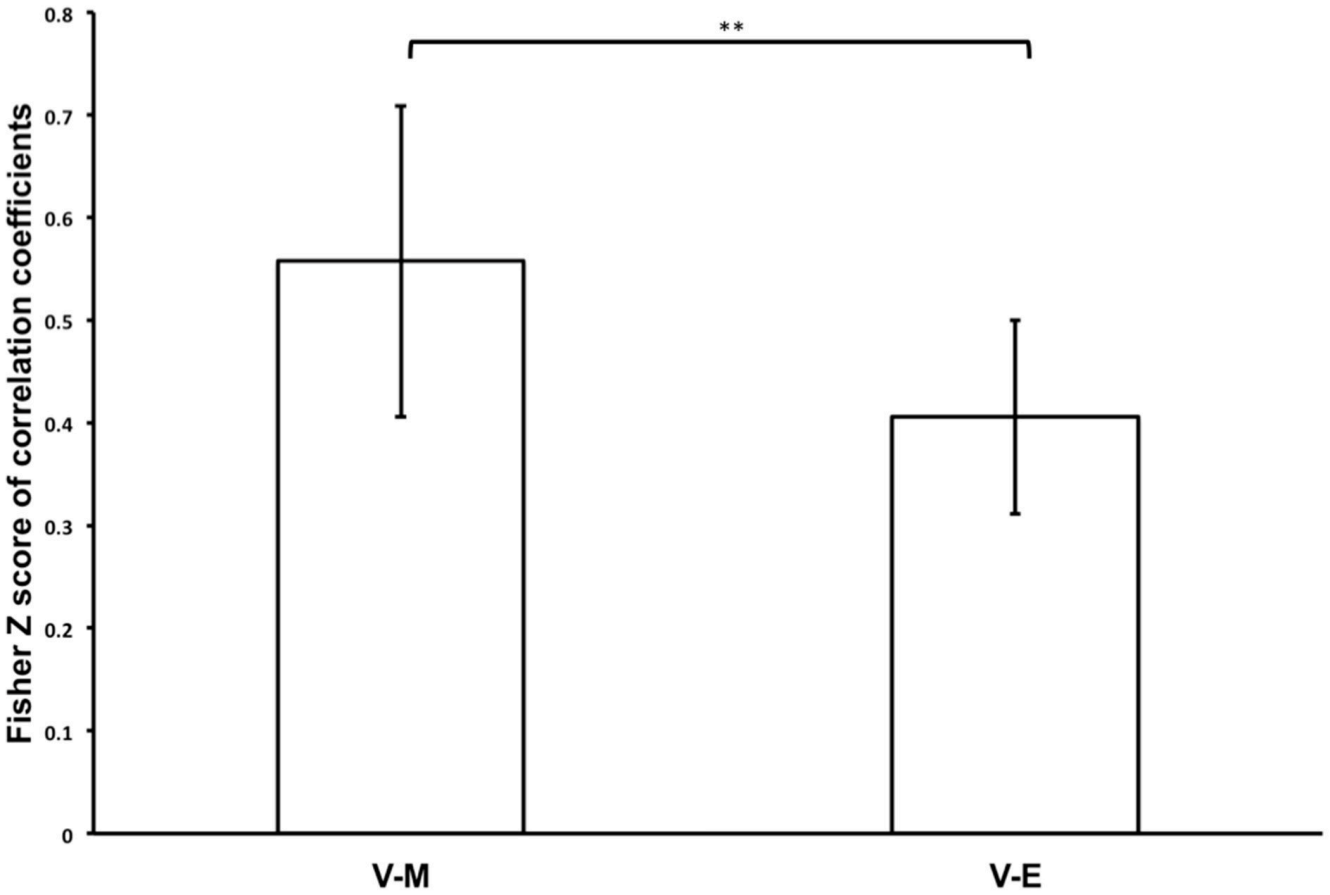
Bar graph showing mean Fisher Z-transforms of V-M and V-E correlations for the whole participant sample (^**^p < 0.01). Error bars denote 95% confidence intervals.

We further considered that comparisons of regional magnitudes of task-related neural responses might only provide partial explanation for the degree of whole-brain correspondences of neural processing during Value, Mathematical, and Emotion tasks. Thus, we performed a spatial correlation analysis to determine whether the pattern of neural activity across voxels in the whole-brain during the Value task processing was more similar to the Mathematical or Emotion tasks. We found that Mathematical and Emotion task spatial activities were significantly correlated with the Value task during the choice phase [V-M: mean Z_r_ (SD) = 0.571 (0.352), *p* < 0.001; V-E: mean Z_r_ (SD) = 0.426 (0.221), *p* < 0.001; Figure 5]. Critically, a paired *t*-test showed that the correlations between Value and Mathematical tasks were significantly greater than the correlations between Value and Emotion tasks across participants [*t*_(19)_ = 3.06, *p* < 0.01].

### Feedback phase brain responses

**Figure 6:**
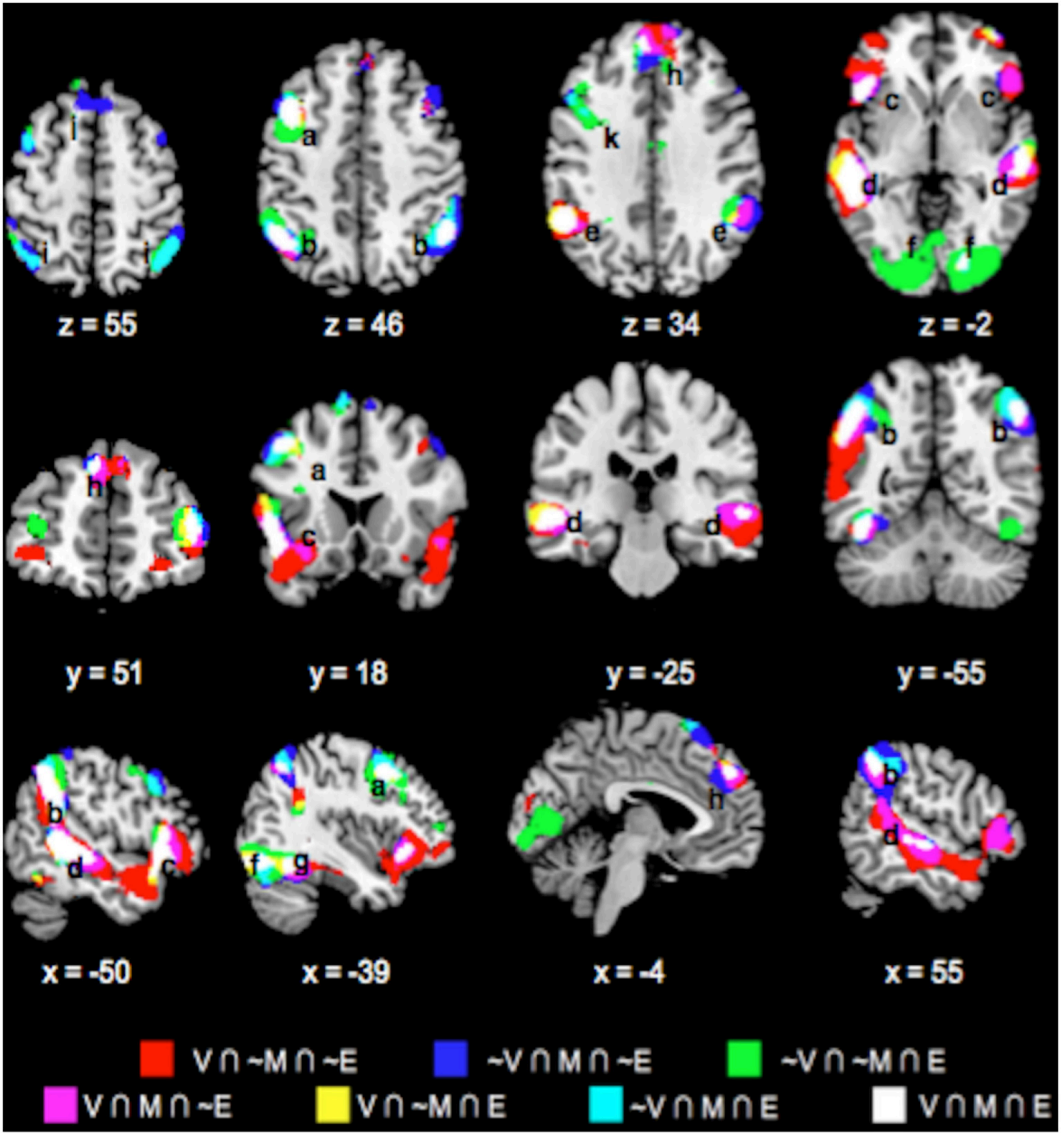
**Overlay maps showing key brain regions with significant positive task responses during the Feedback phases of the Value (V), Mathematical (M), and Emotion (E) tasks, as well as their overlapping areas**. Region labels: (a) Bilateral Angular Gyrus, (b) Bilateral Medial Superior Frontal Gyri (BA8), (c) Bilateral Inferior Parietal Lobule, (d) L Middle Frontal Gyrus (BA9), (e) Bilateral Medial Superior Frontal Gyri (BA9), (f) Bilateral Supramarginal Gyri, (g) L Precentral gyrus, (h) Bilateral Middle Temporal Gyri, (i) Bilateral Inferior Frontal Gyri (BA45/47), (j) Bilateral Inferior Occipital Gyri, (k) R Middle Frontal Gyrus (BA46), (l) L Fusiform Gyrus. Voxel significance threshold was set at *p* < 0.001 with cluster size 50.

**Figure 7:**
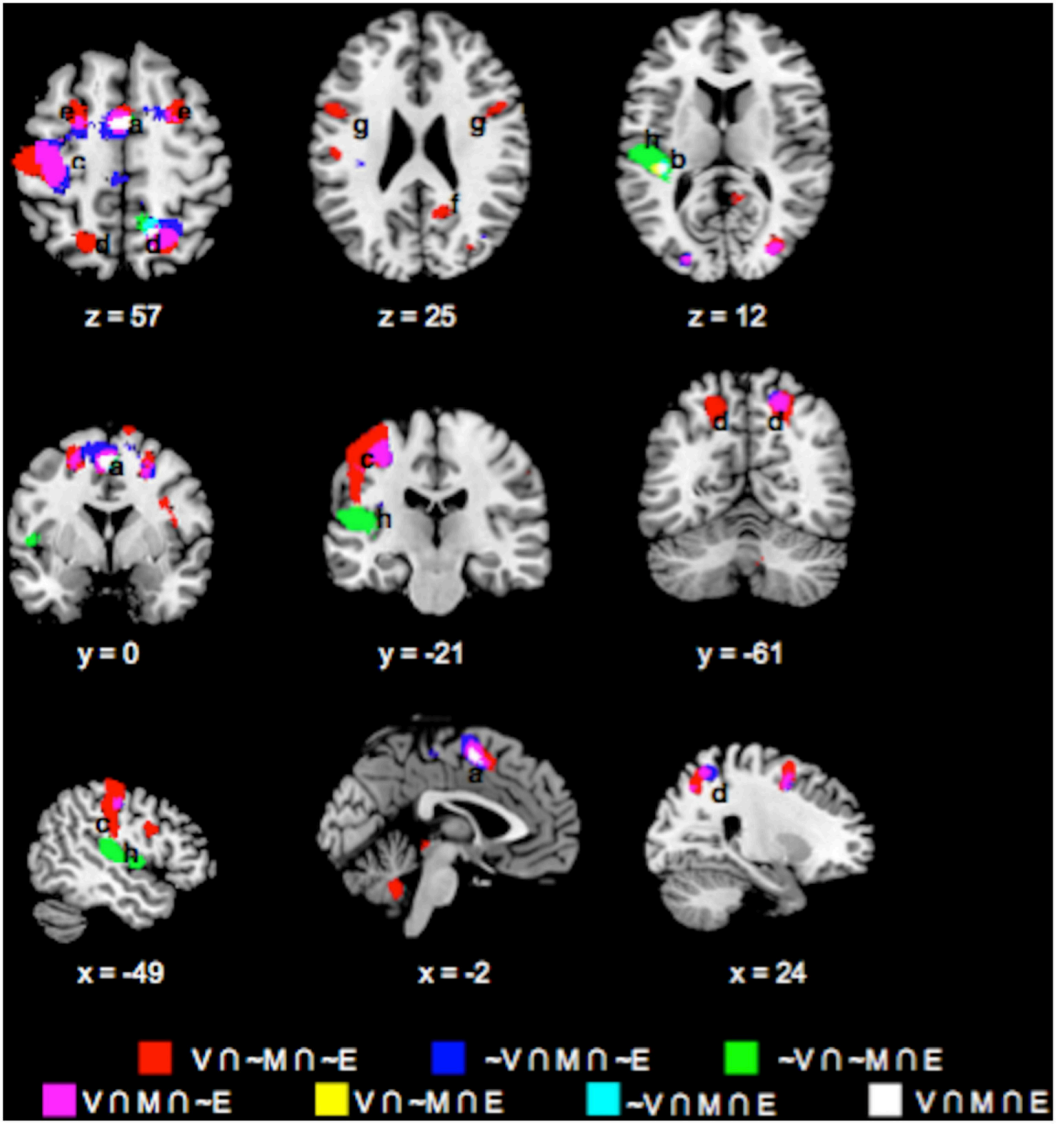
**Overlay maps showing brain regions with significant negative task responses during the Feedback phases of the Value (V), Mathematical (M), and Emotion (E) tasks, as well as their overlapping areas**. Region labels: (a) Bilateral Supplementary Motor Area, (b) R Superior Parietal Lobule, (c) L Postcentral Gyrus, (d) Bilateral Superior Frontal Gyri, (e) R Precuneus, (f) Bilateral Precentral Gyri, (g) L Superior Temporal Gyrus, (h) L Rolandic Operculum, (i) R Middle Frontal Gyrus (BA 6). Voxel significance threshold was set at *p* < 0.001 with cluster size 50.

**Figure 8:**
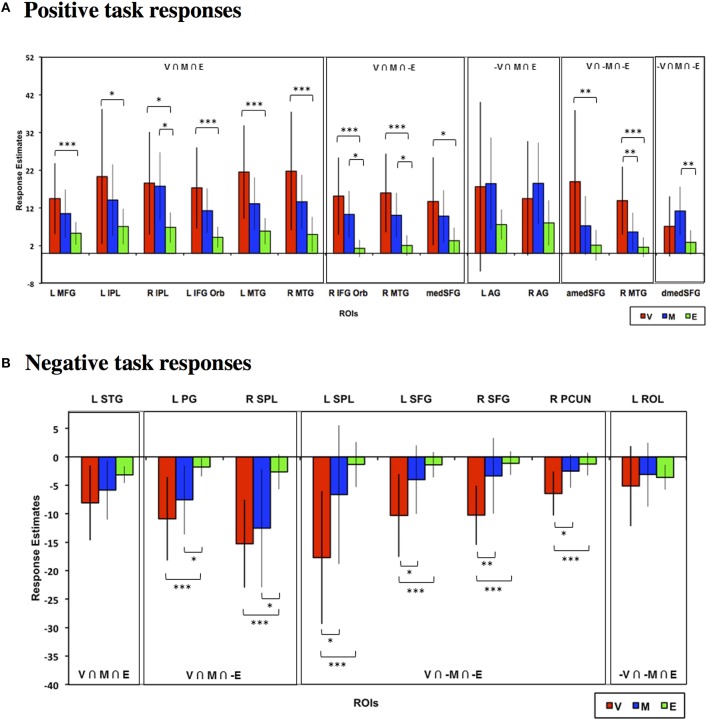
**Mean estimated neural responses during the feedback phases of the Value (V), Mathematical (M), and Emotion (E) tasks in ROIs based on the conjunction analyses. Error bars denote 95% confidence intervals**. Plots are shown for ROIs showing **(A)** Positive and **(B)** negative task responses (^*^p < 0.05, ^**^p < 0.01, ^***^p < 0.001). Region labels: L IFG Oper, Left Inferior Frontal Gyrus Pars Opercularis; R IFG Oper, Right Inferior Frontal Gyrus Pars Opercularis; L MFG, Left Middle Frontal Gyrus; L SPL, Left Superior Parietal Lobule; R SPL, Right Superior Parietal Lobule; L Pt, Left Putamen; R Pt, Right Putamen; L Thal, Left Thalamus; medSFG, Medial Superior Frontal Gyrus; L IFG Orb, Left Inferior Frontal Gyrus Pars Orbitalis; L IFG Tri, Left Inferior Frontal Gyrus Pars Triangularis; R IFG Tri, Right Inferior Frontal Gyrus Pars Triangularis; L Ins, L Insula (for positive task response: −30, 18, 3; for negative task responses: −42, 18, 0), R Thal, Left Thalamus; L SMG, Left Supramarginal Gyrus; R SMG, Right Supramarginal Gyrus; R AG, Right Angular Gyrus.

We also evaluated whether task differences would also be associated with differential neural engagement during feedback. We performed the same conjunction analysis on the mean feedback phase responses across the Value, Mathematical, and Emotion tasks (Figures 6, 7, Table 2). For positive responses during feedback, common significant activities across all three tasks were present in bilateral medial, inferior, and middle frontal, inferior parietal, middle temporal, and occipital areas. Unlike during the choice phase, we observed more expansive involvement of the inferior frontal and middle temporal regions for the Value than Mathematical or Emotion tasks during feedback (Figures 6H,I). For negative responses during feedback, only the supplementary motor and left superior temporal/posterior insula areas showed common significant responses across all three tasks (Figures 7A,G). Again, more expansive involvement was observed for the Value than other tasks in bilateral precentral and left postcentral and superior parietal regions (Figures 7B,C,F). ROI analyses corroborated greater magnitudes of neural responses during the feedback phases of Value than Mathematical or Emotion tasks across several brain areas (Figure 8). Spatial correlation analysis of feedback phase responses yielded similar results as the choice phase responses. Specifically, both Mathematical and Emotion task spatial activities were significantly correlated with the Value task during the feedback phase [V-M: mean Z_r_ (SD) = 0.546 (0.250), *p* < 0.001; V-E: mean Z_r_ (SD) = 0.402 (0.216), *p* < 0.001] with the correlations between Value and Mathematical tasks being greater than between Value and Emotion tasks [*t*_(19)_ = 2.71, *p* < 0.05]. Overall, our findings point to more extensive processing of feedback information during Value than Mathematical or Emotion tasks, consistent with a stronger sense of subjective value involved in the Value task.

**Table 2:** **Peak MNI coordinates with Brodmann's Areas (BA) and minimal t-statistic of conjunction analyses of brain regions with significant common or unique responses during the feedback phases of the Value (V), Mathematical (M), or Emotion (E) tasks**.

**Conjunction**	**Regions**	**BA**	***x***	***y***	***z***	***t***
V ∩ M ∩ E	L Middle Temporal Gyrus	21	−54	−28	−2	7.29
	R Inferior Parietal Lobule	40	52	−51	42	4.76
	L Inferior Frontal Gyrus Pars Orbitalis	47	−46	24	−6	5.51
	L Middle Frontal Gyrus	9	−44	14	45	5.01
	R Middle Temporal Gyrus	21	58	−27	−2	3.96
	L Supramarginal Gyrus	42	−52	−46	25	5.27
	R Lingual Gyrus	18	20	−87	−8	7.08
	L Lingual Gyrus	18	16	−87	−12	5.44
	L Inferior Occipital Gyrus	19	−39	−78	−11	4.56
	L Fusiform Gyrus	37	−40	−54	−15	5.23
	R Superior Temporal Gyrus	22	62	−16	−3	4.39
	L Superior Medial Frontal Gyrus	9	−4	48	36	4.23
	L Rolandic Operculum	48	−39	−30	12	−4.19
	L Supplementary Motor Area	6	−3	0	54	−4.71
V ∩ M ∩~E	R Inferior Frontal Gyrus Pars Orbitalis	47	48	26	−5	5.9
	R Middle Temporal Gyrus	22	56	−15	−8	6.09
	L Superior Medial Frontal Gyrus	9	−2	45	36	4.15
	R Superior Medial Frontal Gyrus	9	−4	54	34	4.02
	L Inferior Frontal Gyrus Pars Triangularis	47	−44	26	0	5.56
	L Insula	47	−30	20	−14	4.74
	L Middle Temporal Gyrus	20	−54	−22	−11	5.35
	L Fusiform Gyrus	37	−42	−49	−21	4.98
	R Angular Gyrus	40	57	−48	37	4.38
	L Postcentral Gyrus	3	−35	−25	49	−5.24
	R Superior Parietal Lobule	5	18	−58	56	−4.94
	R Middle Occipital Gyrus	19	34	−78	18	−4.22
	L Supplementary Motor Area	6	−2	2	58	−4.79
V ∩~M ∩ E	L Fusiform Gyrus	18	−24	−85	−12	5.72
	L Lingual Gyrus	18	−28	−88	−17	4.51
	L Middle Temporal Gyrus	21	−62	−24	−2	5.43
	L Angular Gyrus	41	−46	−48	25	5.19
	L Supramarginal Gyrus	40	−51	−45	36	4.44
	L Inferior Parietal Lobule	40	−46	−49	42	4.14
	L Superior Temporal Gyrus	38	−52	15	−15	5.01
	R Inferior Occipital Gyrus	19	42	−84	−14	4.99
	R Middle Frontal Gyrus	46	39	52	7	4.09
	L Superior Temporal Gyrus	41	−42	−31	12	−4.34
~V ∩ M ∩ E	R Angular Gyrus	39	44	−58	54	4.97
	L Inferior Parietal Lobule	40	−51	−54	51	4.31
	L Inferior Occipital Gyrus	18	−18	−90	−8	4.94
	L Inferior Temporal Gyrus	37	−44	−46	−12	4.82
	L Fusiform Gyrus	37	−39	−54	−9	4.19
	L Middle Frontal Gyrus	44	−40	9	37	4.52
	R Inferior Occipital Gyrus	18	28	−87	−14	4.38
	L Superior Frontal Gyrus	8	−12	26	63	4.27
	L Supplementary Motor Area	6	−8	18	67	3.94
	L Middle Frontal Gyrus	6	−40	8	51	4.25
V ∩~M ∩~E	R Superior Frontal Gyrus	10	18	64	15	3.55
	R Superior Medial Frontal Gyrus	9	12	44	33	3.51
	R Middle Temporal Gyrus	21	62	−21	−11	6.10
	L Inferior Frontal Gyrus Pars Triangularis	47	−42	32	0	6.46
	L Fusiform Gyrus	37	−38	−33	−18	4.68
	R Inferior Frontal Gyrus Pars Orbitalis	45	54	34	−6	4.51
	L Middle Orbital Frontal Gyrus	11	−26	48	−11	3.57
	R Middle Orbital Frontal Gyrus	47	32	57	−5	4.03
	L Superior Parietal Lobule	7	−18	−66	46	−4.71
	L Superior Frontal Gyrus	8	−26	6	61	−4.57
	R Superior Frontal Gyrus	6	26	5	61	−5.11
	R Precuneus	23	16	−54	24	−4.18
	L Postcentral Gyrus	4	−44	−18	58	−5.24
	R Middle Occipital Gyrus	19	33	−75	18	−4.44
	R Supplementary Motor Area	6	2	6	57	−3.94
	L Supplementary Motor Area	32	−3	11	51	−4.17
~V ∩ M ∩~E	L Dorsal Superior Medial Frontal Gyrus	8	−6	27	60	5.26
	R Dorsal Superior Medial Frontal Gyrus	8	8	29	60	4.90
	R Angular Gyrus	39	48	−60	45	5.18
	L Superior Medial Frontal Gyrus	32	−6	41	33	5.18
	R Superior Medial Frontal Gyrus	9	2	41	42	3.81
	L Inferior Parietal Lobule	40	−42	−61	55	4.98
	L Angular Gyrus	7	−38	−66	49	4.00
	L Middle Temporal Gyrus	48	−48	−22	−3	4.58
	L Fusiform Gyrus	37	−34	−54	−12	4.91
	R Middle Frontal Gyrus	46	48	54	4	4.62
	R Inferior Frontal Gyrus Pars Orbitalis	47	45	28	−2	4.57
	L Superior Frontal Gyrus	6	−20	−3	55	−3.58
	L Supplementary Motor Area	6	−6	−1	61	−6.07
	R Superior Frontal Gyrus	6	20	5	49	−4.68
	L Postcentral Gyrus	3	−27	−27	49	−4.92
	R Precuneus	5	16	−57	62	−5.60
~V ∩~M ∩ E	R Inferior Occipital Gyrus	18	26	−90	−3	8.87
	L Precentral Gyrus	6	−42	5	43	5.58
	L Inferior Parietal Lobuls	40	−51	−45	40	4.98
	R Middle Frontal Gyrus	45	39	50	10	4.70
	L Middle Frontal Gyrus	44	−39	21	40	4.64
	R Angular Gyrus	40	45	−48	37	4.61
	R Supramarginal Gyrus	48	48	−40	31	4.22
	R Inferior Frontal Gyrus Pars Triangularis	45	40	32	28	4.41
	R Superior Medial Frontal Gyrus	32	10	36	37	4.34
	L Inferior Parietal Lobule	7	−30	−54	43	4.08
	L Rolandic Operculum	48	−51	−18	9	−5.25

L, Left Hemisphere; R, Right Hemisphere.

### Analysis based on a priori rois

**Figure 9:**
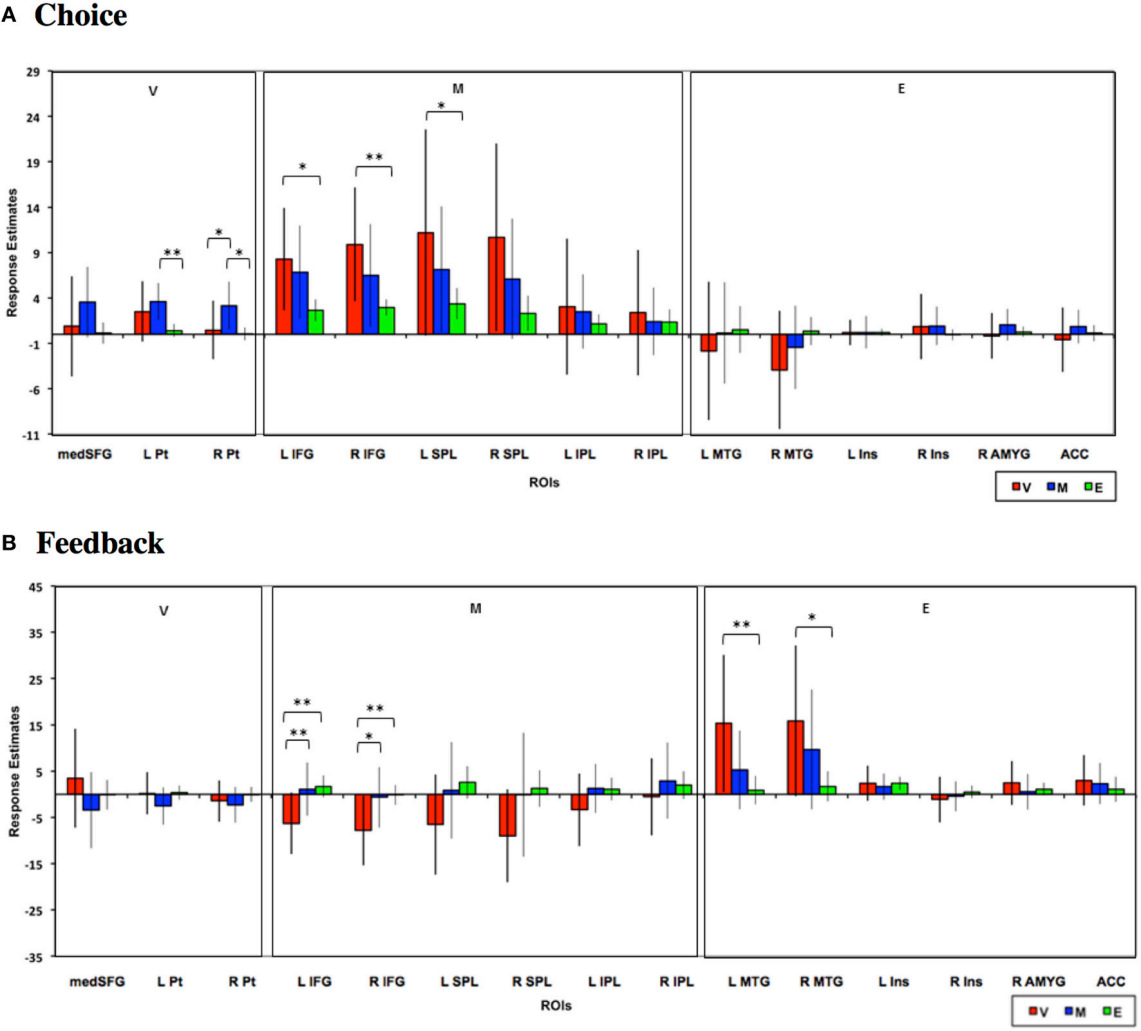
**Mean estimated neural responses during the (A) choice and (B) feedback phases of the Value (V), Mathematical (M), and Emotion (E) tasks in ROIs based on meta-analytic studies that identified Value, Mathematical, and Emotion processing ROIs (see Materials and Methods)**. Error bars denote 95% confidence intervals (^*^p < 0.05, ^**^p < 0.01). Region labels: medSFG, Medial Superior Frontal Gyrus; L Pt, Left Putamen; R Pt, Right Putamen; L IFG, Left Inferior Frontal Gyrus; R IFG, Right Inferior Frontal Gyrus; L SPL, Left Superior Parietal Lobule; R SPL, Right Superior Parietal Lobule; L IPL, Left Inferior Parietal Lobule; R IPL, Right Inferior Parietal Lobule; L MTG, Left Middle Temporal Gyrus; R MTG, Right Middle Temporal Gyrus; L Ins, Left Insula; R Ins, Right Insula; R AMYG, Right Amygdala; ACC, Anterior Cingulate Cortex.

Finally, we also evaluated choice and feedback phase responses in *a priori* ROIs reported in previous meta-analyses to be involved in valuative, mathematical, or emotional processing (see Materials and Methods). As can be seen in Figure 9, neural responses in these ROIs generally replicated the above findings based on the conjunction analysis ROIs. Specifically, bilateral putamen ROIs were predominantly involved in the Mathematical task and inferior frontal and superior parietal ROIs showed significantly higher responses during Value than Emotion tasks. Feedback responses were also of greater magnitude in the Value than other tasks in the frontal, parietal, and temporal ROIs. We note, however, that because these were *a priori* ROIs defined from meta-analyses, the neural responses evaluated in these ROIs are likely not the most representative of responses to our tasks in general.

## Discussion

This present study demonstrated the spatial neural correlates of where mathematical and emotional processing overlap with or are unique from processes engaged during value-based decision-making. We adopted minimal expectations on which brain areas would be involved during the Value, Mathematical, and Emotion tasks. With this neutral approach, we found that these three tasks commonly involved lateral frontal, parietal, motor, somatosensory and visual areas. Mathematical and valuative processing further overlapped in bilateral striatum. Emotional and valuative processing overlapped in areas within parietal, motor, and sensory cortex that also shared involvement with mathematical processing, albeit in different areas within the same cortical structures. Emotional processing involved the most expansive area of neural activity amongst the three tasks. Valuative processes did not show clear specialized areas apart from mathematical or emotional processes. Overall, neural activity engaged during valuative processing was more similar to mathematical than emotional processing. These results suggest a more objective strategy in our sample of young adult human participants during valuative decision-making under the neutral context instantiated in our task.

### Common cortical engagement across value, mathematical, and emotion tasks

As mentioned, it is not surprising that the use of similar stimuli format and stimuli-response mappings resulted in common recruitment of the visual and motor systems across tasks in our study. Common involvement of the lateral frontal and parietal regions across tasks might also be expected due to recruitment of general attention and working memory processes to select and maintain stimuli representations for decision processes to operate (Naghavi and Nyberg, 2005). Fronto-parietal activity has further been implicated in deliberative processing associated with planning and decision-making (Andersen and Cui, 2009).

That these areas involved in valuative and mathematical processing were also engaged during emotional processing may indicate that decisions based on affective information also involve some minimal level of valuative deliberation (Farrell et al., 2014). Besides, how frontal and parietal regions were engaged across the Value, Mathematical, and Emotion tasks may reflect a general neural mechanism for affective and deliberative processing when decisions of any type are to be made. Specifically, the Emotion task recruited widespread areas throughout the cortex with lower but less variable neural activity levels. Also, the Value and Mathematical tasks evoked activity within the same network of regions but in more focal and restricted loci with higher and more variable neural activity levels. Based on this, we speculate that deliberation selectively increases activity of core neural regions relevant for immediate tasks whereas affective computations proceed at lower levels of neural activity. This pattern of more selective deliberative than affective brain involvement was observed even under easier conditions (Supplementary Figures 1–3). Importantly, more selective neural recruitment during deliberation simultaneously suppresses adjacent neural activity resulting in a more dynamic range of neural responses in the network. This notion is in line with neurocomputational models of dopamine action on cortical, particularly frontal, activity that suggest increasing neural signal gain via modulating dopamine levels in the cortex leads to greater signal-to-noise ratio and capacity of the neural network to distinctively represent different information states (Li et al., 2010). Future more targeted studies investigating the specific contributions of the observed focal and non-focal frontal and parietal regions toward decision behaviors will be required to verify these speculations.

### Value and mathematical processing

The involvement of the striatum during both Value and Mathematical but not Emotion tasks highlights the role of this subcortical region during deliberative cognitive processing. Supporting evidence comes from dopamine-related studies where participants with higher striatal levels of the neurotransmitter, associated with higher striatal activity, have better cognitive performance (Vernaleken et al., 2007; Cools et al., 2008). In addition, higher striatal presynaptic levels of dopamine is associated with greater disposition to make model-based rather than model-free choices Deserno et al. (2015). Note that our data do not contravene striatal contributions to model-free behaviors in many decision contexts (Daw et al., 2011). However, at least in our experimental context where value information during the choice phase of the Value task was explicitly presented without affective biases, participants engaged neural processes during valuative processing that were more similar to mathematical than emotional processing, and striatal regions were a part of where these processes converged. We suggest that the striatal activity observed during the Value and Mathematical tasks may reflect the integration and re-integration of valuative or numerical magnitude signals, respectively, arriving from other brain areas over several iterations to progressively arrive at a decision. Studies involving greater sensitivity to temporal information on neural processing may help characterize striatal involvement by evaluating whether its neural processing might be model-free at first but become model-based later on under certain task demands.

We note that both Value and Mathematical tasks similarly involved numerical calculation (product of top and bottom numbers). As such, cognitive differences between these two tasks, if any, should be associated with greater processing of subjective incentive value in the former that is not entirely absent but reduced in the latter task. Indeed, in our experimental design, the predetermined “correct” answers in the Value task were configured to be stochastically favorable to the higher expected values and less favorable to the lower expected values. In contrast, correct answers in the Mathematical task were always deterministically based on the numerical result. In addition, participants always performed the Value task first with instruction to select stakes to maximize overall outcome, followed by the Mathematical task with instruction to calculate and choose the higher numerical result. Critically, our results showed that the Value task suppressed supramarginal and dorsal medial frontal responses more than the Mathematical task during the choice phase. Also, the Value but not Mathematical task evoked significantly higher responses than the Emotion task in lateral frontoparietal areas. Finally, feedback brain responses were also more extensive for the Value than the Mathematical task. Thus, despite the task and brain response similarities between Value and Mathematical tasks, there were still distinctive patterns of engagement likely related to differences in the sense of subjective value.

### Emotional vs. valuative processing

While showing common involvement with valuative processing in several brain areas, emotional processing additionally evoked responses in bilateral thalamus, the more anterior portion of bilateral frontal areas, and left insula that were less involved during the Value and Mathematical tasks. Along with the brain regions commonly involved across the three tasks, these additional regions have all been implicated in the processing of emotional features (Phan et al., 2002). We attribute the extensive thalamic activity during the Emotion task to its role in relaying sensory visual information about facial emotion expressions to the rest of the cortex in order to meet task demands. By contrast, the Value and Mathematical tasks emphasized the text stimuli that were physically simpler than face emotion stimuli and may not have required as much sensory visual processing. We note that the left posterior thalamus was also somewhat responsive during the Mathematical task, which we interpret as due to the greater task difficulty of arithmetic calculations inducing increased levels of motor control (Bosch-Bouju et al., 2013).

Interestingly, the modulation of emotional processing by cognitive demand has been reported to be minimal in the thalamus but more evident in insula and lateral frontal function (Phan et al., 2002). The insula has been regarded as an interoceptive cortex involved in representing internal emotional and bodily states (Paulus and Stein, 2006; Craig, 2009). In addition, lateral frontal regions have been implicated in emotion perception and regulation (Banks et al., 2007). In support of the above distinction between thalamic, frontal, and insula contributions to emotional processing, we found the Emotion task engaged the thalamus with minimal involvement during the Value and Mathematical tasks. In addition, although the Value and Mathematical tasks yielded less extensive neural activity in the insula and anterior frontal areas than the Emotion task, nearby subsets of other areas within the same brain structures also responded during these former tasks. Overall, our findings suggest that decision processing based on expected value or arithmetic quantities relied minimally on brain areas involved in sensory visual processes during decision processing based on emotional facial expressions. However, value and mathematical processing may require interoceptive and regulatory processing in insula and frontal areas, respectively, albeit in a more focal manner than during emotional processing.

### Task-negative responses

Lower neural activities relative to task resting baselines, or deactivation, were observed in the medial frontal, bilateral supramarginal, right angular, left inferior frontal, and insula regions. This finding is consistent with reports of these brain regions as being part of the default-mode network (DMN) that is more active during baseline than task (Raichle et al., 2001). The DMN is involved in introspective cognitive processes such as the direction of attention to one's present emotional or physical state, appraisal of self-identity or personality, and thinking about one's past or future (Andrews-Hanna et al., 2010). These introspective processes are distinct from the processing of exogenous stimuli so that greater suppression of DMN neural responses have been linked to better task performances and other external goal directed behaviors (Gusnard and Raichle, 2001; Hester et al., 2004). Critically, being “default,” DMN neural deactivation responses when processing exogenous stimuli are understood to be general and reliable across many different kinds of tasks and contexts. However, we found that there were quite different patterns of neural deactivations across the Value, Mathematical, and Emotion decision tasks in the above DMN regions identified in our study. In general, the Value task induced the most neural deactivation across these regions whereas the Emotion task elicited almost no deactivation at all with intermediate deactivations during the Mathematical task. At the very least, there was minimal overlap of brain regions showing common neural deactivation between the three tasks. We suggest that this pattern of responses might reflect critical inhibition of irrelevant introspective processes during valuative and mathematical processing to support performance for these more objective tasks, particularly in the left insula (Damasio et al., 2000). By contrast, introspective processes may be more relevant during processing of facial expressions during the Emotion task such that DMN responses were generally not suppressed.

### Contributions of attention, difficulty, and mathematical competency

In terms of the task design, the Emotion task directed attention to emotional faces whereas faces could be neglected in the other two tasks, albeit a neutral face was still shown, and attention directed instead to simple text stimuli. In addition, the Value and Mathematical tasks shared more surface features than the Emotion task. Thus, our aim to implement a less biased decision context likely resulted in mathematical processes playing a more prominent role in the Value task compared with emotional processes. Further, greater similarity in brain activity between Value and Mathematical tasks may be due to the common attention to the text stimuli in the tasks. In addition, lower similarity between Value and Emotion tasks may arise from differential attention to text and faces between the tasks, respectively. Nevertheless, in addition to the abovementioned differences related to additional processing of subjective value in the Value task, we highlight that the Mathematical task still yielded a uniquely involved region during the choice phase in the medial superior frontal area that did not respond during the Value (or Emotion) task. Also, although there was common striatal involvement in valuative and mathematical choice processing, striatal voxels responding during the Value task were a subset of those responding to the Mathematical task so that the two tasks still evinced clear differences in functional responses.

Unlike the brain imaging data, behavioral accuracy and response time data showed greater similarity between the Value and Emotion tasks than between the Value and Mathematical tasks. Specifically, the slower response times during the Mathematical task reflected greater difficulty and attentional demands than the other two tasks. Taken together, these behavioral findings suggest differential attention to the text or face stimuli cannot sufficiently account for the different similarity in brain responses between valuative and mathematical, and between valuative and emotional processing. It is possible that, because of the stochastic nature of outcomes, the similarity of the behavioral data between Value and Emotion tasks stemmed from participants guessing in both tasks. However, we highlight that given the above chance performance for the easier Congruent and Incongruent IV conditions of the Value task, guessing was minimal for the task and participants were likely attempting to use the given information to compute expected values. Thus, the behavioral similarity between Value and Emotion tasks is likely a surface result that does not relate to the functional similarity between Value and Math tasks. Although response times were longer during the Math than Value task, the brain regions involved were more similar than for the Emotion task, which we deem as the more important comparison. In this case, behavioral data offered a limited, even conflicting view about the underlying processes, and brain data was more revealing.

We considered the role of mathematical competency in modulating neural and behavioral responses in the Value and Mathematical tasks. We highlight that performance in the Mathematical task was better than the Value task for the same level of calculation difficulty (compare Supplementary Table 1, Incongruent III for Value to Incongruent I for Mathematical tasks, and Incongruent IV for Value to Incongruent II for Mathematical tasks). It is difficult to attribute this behavioral result to mathematical competency. Rather, we argue that this performance change was because the same numerical content was framed under a stochastic context in the Value task, so that participants reduced their rate of optimal choices instead of adhering to pure calculation-based decisions. In addition, we designed our study to acquire data in a within-subject manner so that individual differences in the effect of mathematical competency on Math and Value task performance and processing would be equated for each participant. Finally, we found similar spatial distribution of engaged brain regions when the conjunction analysis was based on the easiest Math and Value conditions (Supplementary Figures 1–3). Taken together, these suggest that mathematical competency had negligible contribution to the findings in our experiment as it was designed.

### Contributions of social processing

Apart from facial emotional expressions our Emotion task may also have involved some element of social trust or preference judgment. We note that in our study, age and sex were balanced for the facial stimuli used. Thus, differences in the emotional valences of the faces were the primary and, by design, most accurate feature participants could use to make their decisions to obtain subjective economic incentives. Even if participants made decisions based more on a sense of social preference, we submit that a large part of this likely stemmed from the differences in emotional expressions. That is, in the Emotion task, affective emotional information must still be used to inform social judgments, if that is the strategy adopted. Thus, while it is difficult to separate emotional content from social meaning in the Emotion task, we suggest that there is no need to do so in our present study as both involve the use of emotional information to attain personal economic prospects. Further detail on the neural processes underlying differences or conflict between social and economic incentives would be a critical topic for future studies (although see Farrell et al., 2014).

## Conclusion

This present study identified the neural loci where cognitive and affective processes contribute toward value-based decision-making. Overall, neural responses during valuative processing were more similar to mathematical processing than emotional processing, particularly. This similarity between valuative and mathematical processing is characterized by common striatal activity and more focal engagement of brain regions already involved in automatic affective processing. Thus, our sample of participants demonstrated objective strategy by adopting more mathematical rather than affective approaches when making decisions based on expected value. It remains for future studies to investigate individual differences in the preference to engage mathematical or affective approaches during valuative decision processing and whether these preferences can be altered.

## Author contributions

JG came up with the idea. CH and JG designed the tasks together. CH did data collection and analysis. CH and JG wrote the paper.

### Conflict of interest statement

The authors declare that the research was conducted in the absence of any commercial or financial relationships that could be construed as a potential conflict of interest.

## Footnotes

^1^We note that although the left thalamus ROI was defined as a region fulfilling the ~V ∩ M ∩ E conjunction, Figure 4A shows that neural responses during V were also significantly above baseline. Further evaluation revealed that there were voxels in this ROI fulfilling V ∩ M ∩ E but these were fewer than 50 in number, preventing it from surviving cluster size correction at the whole-brain analysis level. For this reason, we remain conservative and regard the left thalamus as only significantly responding to the M and E tasks but not V task.

## References

[B1] Andersen R. A. Cui H. (2009). Intention, action planning, and decision making in parietal-frontal circuits. Neuron 63, 568–583. 10.1016/j.neuron.2009.08.028 19755101

[B2] Anderson A. K. Christoff K. Panitz D. De Rosa E. Gabrieli J. D. (2003). Neural correlates of the automatic processing of threat facial signals. J. Neurosci. 23, 5627–5633. Retrieved from: http://www.jneurosci.org/content/23/13/5627.long 12843265 10.1523/JNEUROSCI.23-13-05627.2003 PMC6741280

[B3] Andrews-Hanna J. R. Reidler J. S. Huang C. Buckner R. L. (2010). Evidence for the default network's role in spontaneous cognition. J. Neurophysiol. 104, 322–335. 10.1152/jn.00830.2009 20463201 PMC2904225

[B4] Arsalidou M. Taylor M. J. (2011). Is 2 + 2 = 4? Meta-analyses of brain areas needed for numbers and calculations. Neuroimage 54, 2382–2393. 10.1016/j.neuroimage.2010.10.009 20946958

[B5] Banks S. J. Eddy K. T. Angstadt M. Nathan P. J. Phan K. L. (2007). Amygdala-frontal connectivity during emotion regulation. Soc. Cogn. Affect. Neurosci. 2, 303–312. 10.1093/scan/nsm029 18985136 PMC2566753

[B6] Bartra O. McGuire J. T. Kable J. W. (2013). The valuation system: a coordinate-based meta-analysis of BOLD fMRI experiments examining neural correlates of subjective value. Neuroimage 76, 412–427. 10.1016/j.neuroimage.2013.02.063 23507394 PMC3756836

[B7] Bosch-Bouju C. Hyland B. I. Parr-Brownlie L. C. (2013). Motor thalamus integration of cortical, cerebellar and basal ganglia information: implications for normal and parkinsonian conditions. Front. Comput. Neurosci. 7:163. 10.3389/fncom.2013.00163 24273509 PMC3822295

[B8] Brooks S. J. Savov V. Allzén E. Benedict C. Fredriksson R. Schiöth H. B. (2012). Exposure to subliminal arousing stimuli induces robust activation in the amygdala, hippocampus, anterior cingulate, insular cortex and primary visual cortex: a systematic meta-analysis of fMRI studies. Neuroimage 59, 2962–2973. 10.1016/j.neuroimage.2011.09.077 22001789

[B9] Cannon W. (1932). The Wisdom of the Body. New York, NY: W.W. Norton and Co 10.1097/00000441-193212000-00028

[B10] Cohen Kadosh R. Walsh V. (2009). Numerical representation in the parietal lobes: abstract or not abstract? Behav. Brain Sci. 32, 313–328. 10.1017/S0140525X09990938 19712504

[B11] Cools R. Gibbs S. E. Miyakawa A. Jagust W. D'Esposito M. (2008). Working memory capacity predicts dopamine synthesis capacity in the human striatum. J. Neurosci. 28, 1208–1212. 10.1523/JNEUROSCI.4475-07.2008 18234898 PMC6671420

[B12] Craig A. D. (2009). How do you feel—now? The anterior insula and human awareness. Nat. Rev. Neurosci. 10, 59–70. 10.1038/nrn2555 19096369

[B13] Critchley H. Daly E. Phillips M. Brammer M. Bullmore E. Williams S.. (2000). Explicit and implicit neural mechanisms for processing of social information from facial expressions: a functional magnetic resonance imaging study. Hum. Brain Mapp. 9, 93–105. 10.1002/(SICI)1097-0193(200002)9:2<93::AID-HBM4>3.0.CO;2-Z 10680766 PMC6872127

[B14] Damasio A. R. Grabowski T. J. Bechara A. Damasio H. Ponto L. L. Parvizi J.. (2000). Subcortical and cortical brain activity during the feeling of self-generated emotions. Nat. Neurosci. 3, 1049–1056. 10.1038/79871 11017179

[B15] Daw N. D. Gershman S. J. Seymour B. Dayan P. Dolan R. J. (2011). Model-based influences on humans' choices and striatal prediction errors. Neuron 69, 1204–1215. 10.1016/j.neuron.2011.02.027 21435563 PMC3077926

[B16] Deserno L. Huys Q. J. Boehme R. Buchert R. Heinze H.-J. Grace A. A.. (2015). Ventral striatal dopamine reflects behavioral and neural signatures of model-based control during sequential decision making. Proc. Natl. Acad. Sci. U.S.A. 112, 1595–1600. 10.1073/pnas.1417219112 25605941 PMC4321318

[B17] Ekman P. Friesen W. V. (1977). Facial Action Coding System. Palo Alto, CA: Consulting Psychologists Press; Stanford University.

[B18] Epstein S. Pacini R. (1999). Some basic issues regarding dual-process theories from the perspective of cognitive-experiential self-theory, in Dual-Process Theories in Social Psychology, eds Chaiken S. Trope Y. (New York, NY: The Guilford Press), 462–482.

[B19] Evans J. S. (2003). In two minds: dual-process accounts of reasoning. Trends Cogn. Sci. 7, 454–459. 10.1016/j.tics.2003.08.012 14550493

[B20] Farrell A. M. Goh J. O. White B. J. (2014). The effect of performance-based incentive contracts on system 1 and system 2 processing in affective decision contexts: fMRI and behavioral evidence. Account. Rev. 89, 1979–2010. 10.2308/accr-50852

[B21] Foa E. B. Kozak M. J. (1986). Emotional processing of fear: exposure to corrective information. Psychol. Bull. 99, 20. 10.1037/0033-2909.99.1.20 2871574

[B22] Fusar-Poli P. Placentino A. Carletti F. Landi P. Allen P. Surguladze S.. (2009). Functional atlas of emotional faces processing: a voxel-based meta-analysis of 105 functional magnetic resonance imaging studies. J. Psychiatry Neurosci. 34, 418–432. 19949718 PMC2783433

[B23] Goel V. Dolan R. J. (2003). Explaining modulation of reasoning by belief. Cognition 87, B11–B22. 10.1016/S0010-0277(02)00185-3 12499108

[B24] Gusnard D. A. Raichle M. E. (2001). Searching for a baseline: functional imaging and the resting human brain. Nat. Rev. Neurosci. 2, 685–694. 10.1038/35094500 11584306

[B25] Hester R. Murphy K. Foxe J. J. Foxe D. M. Javitt D. C. Garavan H. (2004). Predicting success: patterns of cortical activation and deactivation prior to response inhibition. Cogn. Neurosci. J. 16, 776–785. 10.1162/089892904970726 15200705

[B26] Kahneman D. Frederick S. (2002). Representativeness revisited: attribute substitution in intuitive judgment. Heurist. Biases 49, 49–81. 10.1017/cbo9780511808098.004

[B27] Li S.-C. Lindenberger U. Bäckman L. (2010). Dopaminergic modulation of cognition across the life span: editorial. Neurosci. Biobehav. Rev. 34, 625–630. 10.1016/j.neubiorev.2010.02.003 20152855

[B28] Liang K.-C. Chen C.-C. (2010). A Study on Standard Stimuli and Normative Responses of Emotion in Taiwan. Retrieved from: http://ssnre.psy.ntu.edu.tw/ssnre/abstract.htm

[B29] Lieberman M. D. (2007). Social cognitive neuroscience: a review of core processes. Annu. Rev. Psychol. 58, 259–289. 10.1146/annurev.psych.58.110405.085654 17002553

[B30] McClure S. M. Laibson D. I. Loewenstein G. Cohen J. D. (2004). Separate neural systems value immediate and delayed monetary rewards. Science 306, 503–507. 10.1126/science.1100907 15486304

[B31] Menon V. Rivera S. M. White C. D. Glover G. H. Reiss A. L. (2000). Dissociating prefrontal and parietal cortex activation during arithmetic processing. Neuroimage 12, 357–365. 10.1006/nimg.2000.0613 10988030

[B32] Naghavi H. R. Nyberg L. (2005). Common fronto-parietal activity in attention, memory, and consciousness: shared demands on integration? Conscious. Cogn. 14, 390–425. 10.1016/j.concog.2004.10.003 15950889

[B33] Nichols T. Brett M. Andersson J. Wager T. Poline J.-B. (2005). Valid conjunction inference with the minimum statistic. Neuroimage 25, 653–660. 10.1016/j.neuroimage.2004.12.005 15808966

[B34] Paulus M. P. Stein M. B. (2006). An insular view of anxiety. Biol. Psychiatry 60, 383–387. 10.1016/j.biopsych.2006.03.042 16780813

[B35] Phan K. L. Wager T. Taylor S. F. Liberzon I. (2002). Functional neuroanatomy of emotion: a meta-analysis of emotion activation studies in PET and fMRI. Neuroimage 16, 331–348. 10.1006/nimg.2002.1087 12030820

[B36] Phelps E. A. LeDoux J. E. (2005). Contributions of the amygdala to emotion processing: from animal models to human behavior. Neuron 48, 175–187. 10.1016/j.neuron.2005.09.025 16242399

[B37] Raichle M. E. MacLeod A. M. Snyder A. Z. Powers W. J. Gusnard D. A. Shulman G. L. (2001). A default mode of brain function. Proc. Natl. Acad. Sci. U.S.A. 98, 676–682. 10.1073/pnas.98.2.676 11209064 PMC14647

[B38] Slotnick S. D. Moo L. R. Segal J. B. Hart J. (2003). Distinct prefrontal cortex activity associated with item memory and source memory for visual shapes. Cognit. Brain Res. 17, 75–82. 10.1016/S0926-6410(03)00082-X 12763194

[B39] Vernaleken I. Buchholz H. G. Kumakura Y. Siessmeier T. Stoeter P. Bartenstein P.. (2007). ‘Prefrontal’ cognitive performance of healthy subjects positively correlates with cerebral FDOPA influx: an exploratory [18F]-fluoro-L-DOPA-PET investigation. Hum. Brain Mapp. 28, 931–939. 10.1002/hbm.20325 17133402 PMC6871482

[B40] Zamarian L. Ischebeck A. Delazer M. (2009). Neuroscience of learning arithmetic—evidence from brain imaging studies. Neurosci. Biobehav. Rev. 33, 909–925. 10.1016/j.neubiorev.2009.03.005 19428500

