# Supplementary material for: Distinct and Overlapping Brain Areas Engaged during Value-Based, Mathematical, and Emotional Decision Processing
Source: Front Hum Neurosci. 2016 Jun 10;10:275. doi: 10.3389/fnhum.2016.00275 (PMC4901075; doi:10.3389/fnhum.2016.00275)
Supplement: Supplementary file 1 [file DataSheet1.PDF]

Supplementary Results (see Supplementary Table 1)

For accuracy in the value task, a one-way repeated-measures ANOVA yielded a significant difference across difficulty levels ($F_{(4,95)}$ = 52.82, p < .001). Tukey post-hoc tests revealed that accuracy was significantly higher for the Congruent level compared with all four Incongruent levels (p < .001 for all comparisons). Accuracies in all four Incongruent levels were not significantly different. For response times, a one-way repeated-measures ANOVA showed no significant difference across difficulty levels ($F_{(4,95)}$ = 0.636, p = .638).

For accuracy in the math task, a one-way repeated-measures ANOVA yielded a significant difference across difficulty levels ($F_{(2,57)}$ = 29.28, p < .001). Tukey post-hoc tests revealed that accuracy was significantly higher for the Congruent level compared with Incongruent I (p < .001) and Incongruent II (p < .001). Accuracies between Incongruent I and Incongruent II were also significantly different (p = .001). For response times, a one-way repeated-measures ANOVA yielded a significant difference across difficulty levels ($F_{(2,57)}$ = 6.949, p < .05). Tukey post-hoc tests revealed that response time was significantly faster for the Congruent level compared with Incongruent I (p < .01) and Incongruent II (p < .05). Response times between Incongruent I and Incongruent II levels were not significantly different (p = .862).
