# Supplementary material for: Distinct and Overlapping Brain Areas Engaged during Value-Based, Mathematical, and Emotional Decision Processing
Source: Front Hum Neurosci. 2016 Jun 10;10:275. doi: 10.3389/fnhum.2016.00275 (PMC4901075; doi:10.3389/fnhum.2016.00275)
Supplement: Supplementary file 2 [file Table1.PDF]

Supplementary Table 1. Performance accuracy and reaction time (RT) by difficulty level in the Value and Mathematical tasks.

Task	Difficulty level	Accuracy Mean (SD)%	Reaction time Mean (SD) ms
Value	[a]Congruent	94.4 (6.8)	1833.9 (354.2)
	[b]Incongruent I	50.6 (10.4)	2037.1 (537.4)
	[c]Incongruent II	56.9 (11.5)	2008.6 (492.4)
	[d]Incongruent III	56.3 (12.0)	2026.0 (468.4)
	[e]Incongruent IV	59.1 (11.1)	2003.8 (415.4)
Mathematical	[a]Congruent	91.5 (7.0)	2230.9 (239.9)
	[f]Incongruent I	64.6 (11.6)	2581.7 (320.7)
	[g]Incongruent II	78.3 (13.0)	2529.0 (364.9)

[a]The magnitudes and probabilities across panels (A&B) were congruent such that both were higher in one panel than the other.

[b]The magnitudes were higher with lower probabilities in one panel. The numbers used were not in multiples of 5 (harder for calculation) and the difference of expected values between panels was smaller than 100 (less distinct difference).

[c]The magnitudes were higher with lower probabilities in one panel. The numbers used were not in multiples of 5 (harder for calculation) and the difference of expected values between panels was larger than 100 (more distinct difference).

[d]The magnitudes were higher with lower probabilities in one panel. The numbers used were in multiples of 5 (easier for calculation) and the difference of expected values between panels was smaller than 100 (less distinct difference).

[e]The magnitudes were higher with lower probabilities in one panel. The numbers used were in multiples of 5 (easier for calculation) and the difference of expected values between panels was larger than 100 (more distinct difference).

[f]The magnitudes were higher with lower probabilities in one panel. The difference of expected values between panels was smaller than 100 (less distinct difference). Manipulation of calculation difficulty (multiple of 5 or not) was not implemented.

[g]The magnitudes were higher with lower probabilities in one panel. The difference of expected values between panels was larger than 100 (more distinct difference). Manipulation of calculation difficulty (multiple of 5 or not) was not implemented.
