# Supplementary figures and images for: Distinct and Overlapping Brain Areas Engaged during Value-Based, Mathematical, and Emotional Decision Processing
Source: Front Hum Neurosci. 2016 Jun 10;10:275. doi: 10.3389/fnhum.2016.00275 (PMC4901075; doi:10.3389/fnhum.2016.00275)

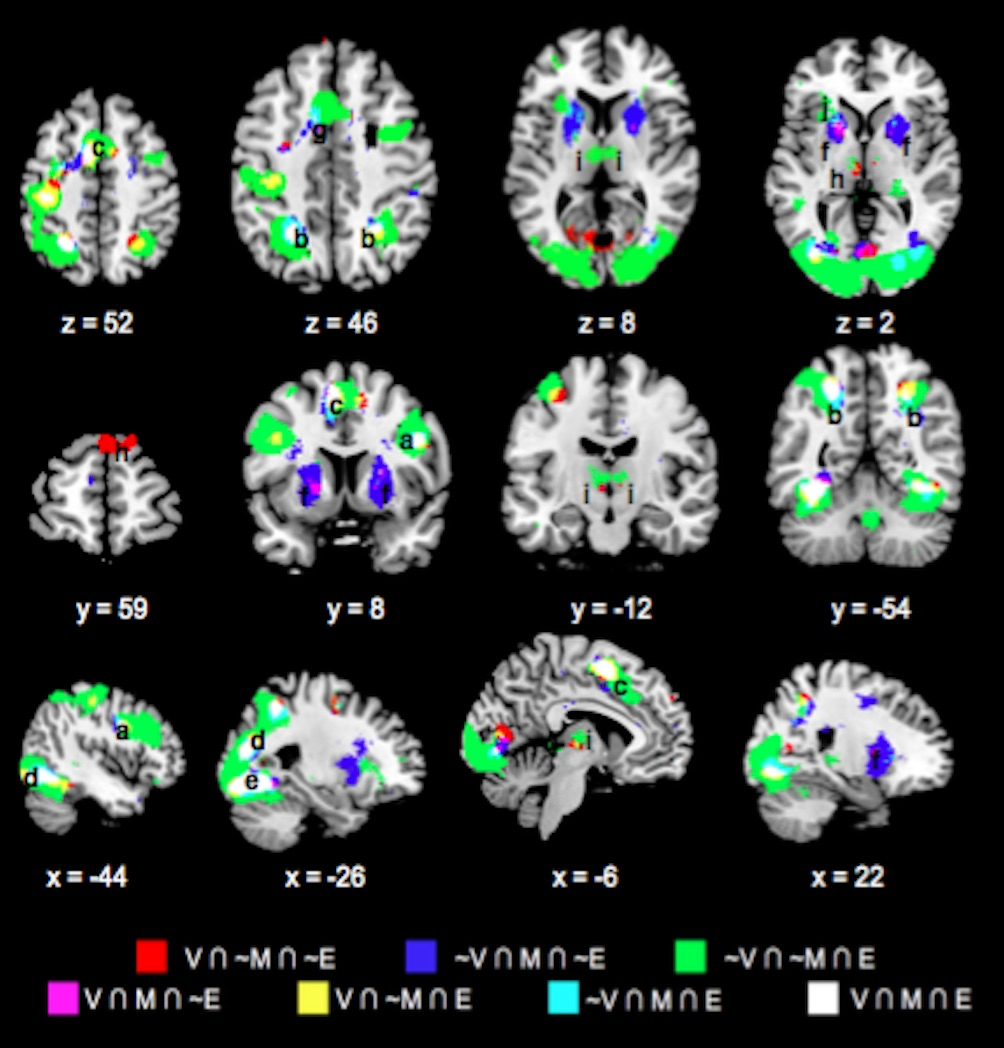

Supplement: Supplementary file 3 [file Image1.TIFF]

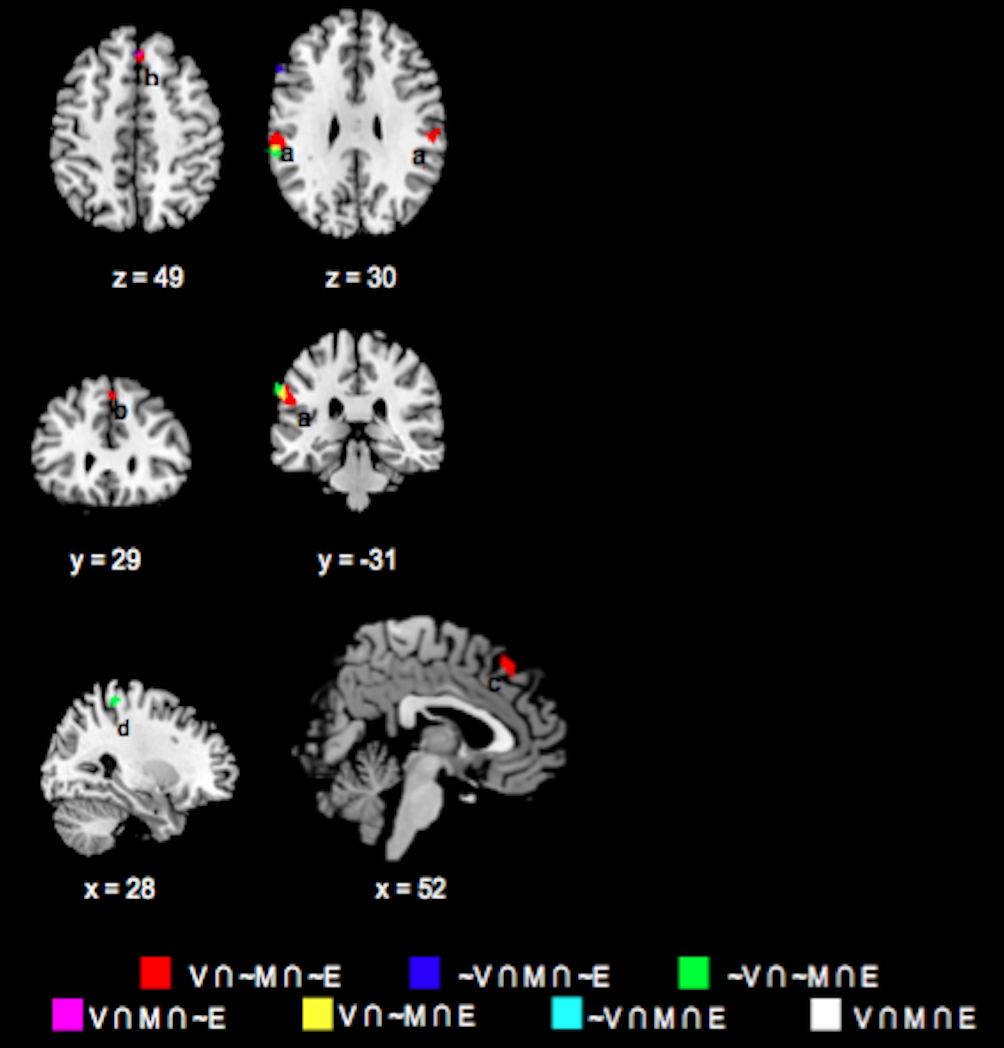

Supplement: Supplementary file 4 [file Image2.TIFF]

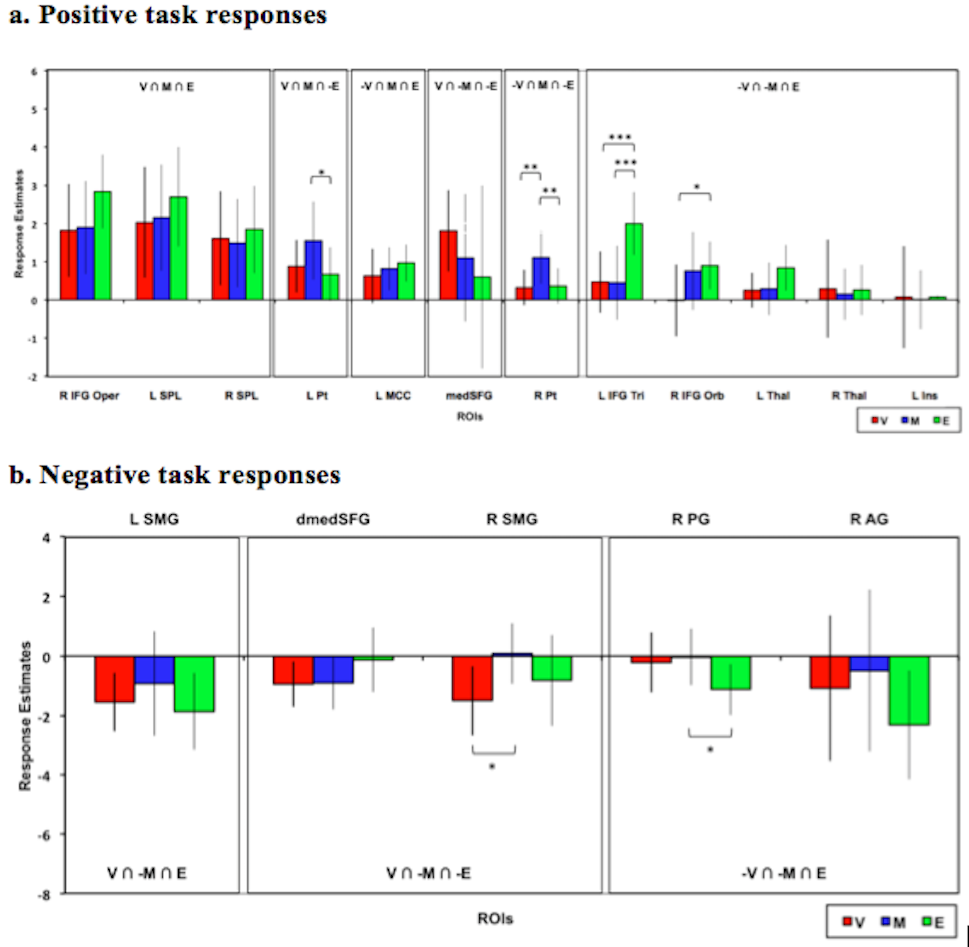

Supplement: Supplementary file 5 [file Image3.TIFF]
